# The junction between the midgut and hindgut co‐localizes with the rectosigmoid junction

**DOI:** 10.1111/joa.70070

**Published:** 2025-11-14

**Authors:** Hui Gao, Nutmethee Kruepunga, Greet Mommen, Susanne E. Köhler, Jill P. J. M. Hikspoors, Wouter H. Lamers

**Affiliations:** ^1^ Department of Anatomy and Embryology Maastricht University Maastricht The Netherlands; ^2^ Department of Anatomy, Faculty of Medicine Kasetsart University Bangkok Thailand

**Keywords:** caudo‐cranial gradient in differentiation, enteric growth, mesenteric width, rectosigmoid junction, splenic flexure

## Abstract

Textbooks locate the junction between the midgut and hindgut where the vascular beds of the superior (SMA) and inferior (IMA) mesenteric arteries meet. In a previous study, we observed that the formation of the midgut corresponded with a pronounced thinning of its dorsal mesentery. We re‐investigated, therefore, the location of the distal boundary of the midgut, making use of 3D reconstructions of serial sections of 36 human embryos between 4 and 13 weeks of development. Using the boundaries of the thin mesentery of the midgut as a criterion, the midgut–hindgut junction corresponds in 10‐week and older foetuses with the rectosigmoid junction. In addition, we established that the 3D orientation of the trunk of the IMA (between its aortic root and first branching node) also identifies the position of the midgut–hindgut junction in the gut. The growth rate of the early colon is exponential, whereas that of the rectum is linear. Initially, the foetal colon has ascending and descending limbs only, of which the descending limb grows fastest. The mesentery of the ascending colic limb adheres to the ventral surfaces of the duodenum, stomach and dorsal pancreas shortly after the hernial return into the abdomen during the 10th week, which rules out an effect of differential growth on the position of the junction. We, therefore, postulate that the rectum is the sole descendant of the embryonic hindgut. The rectum is unique in that its differentiation follows a caudocranial direction. Vascular connections between the perfusion areas of the SMA and IMA expand to form the first colic arterial arcade only at 10 weeks.

## Graphical abstract

At 5 weeks of development, the midgut has formed its primary loop (left). It is well established that the midgut is bounded cranially by the caudal end of the ventral mesentery and the presence of the common bile duct, but its caudal boundary remains to be established. By using the thickness of the mesentery and the position of the trunk of the inferior mesenteric artery (IMA) as criteria, we show that the midgut‐to‐hindgut junction co‐localizes with the rectosigmoid junction. This junction becomes histologically identifiable at 10 weeks of development.

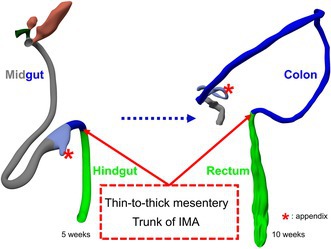

## 1 INTRODUCTION

It is generally accepted that the early embryonic gut consists of three subdivisions: a foregut cranially, a hindgut caudally and a midgut in between. The foregut, in turn, consists of two segments: the pharynx and the caudal foregut. The midgut is the part of the gut that has a roof and two sides, but no floor, because ventrally its lumen is directly continuous with the yolk sac. The caudal foregut, the midgut and the hindgut share a continuous dorsal mesentery, which serves as the gateway for vessels and nerves to the gut. In Carnegie stage 15 (CS15) human embryos (~5 weeks of development), the mesentery extends from the bifurcation of the trachea to the end of the rectum (Hikspoors et al., 2019). The caudal foregut has, in addition, a ventral mesentery. The caudal end of this ventral mesentery harbours the common bile duct and marks the junction between the foregut and midgut. Since the vitelline duct disappears at ~5 weeks of development (CS15; Soffers et al., 2015), this ‘primary’ midgut, with its open connection to the lumen of the yolk sac, ceases to exist, and is superseded by the ‘definitive’ midgut (Soffers et al., 2015; Ueno et al., 2016). The definitive midgut is defined by its junction with the caudal foregut cranially and the hindgut caudally. Only the definitive midgut undergoes looping and temporarily herniates into the umbilical coelom between 5 and 9.5 weeks of development.

Apart from the herniation, the boundary between the definitive midgut and hindgut has not been associated thus far with any non‐controversial anatomical landmark. One of these putative landmarks is the association of segments of the gut with one of the three main ventral abdominal arteries. This concept, present in nearly all textbooks, proposes that the coeliac trunk perfuses the caudal foregut, the superior mesenteric artery (SMA, known as the vitelline artery in early embryos) perfuses the midgut, and the inferior mesenteric artery (IMA) perfuses the hindgut (Douard et al., 2006). In this proposal, the assignment of boundaries to a segment of the gut comes down to identifying the position of the main arterial arcade that connects SMA and IMA. The model appears to be based on Frazer and Robbins (Frazer & Robbins, 1915; Hunter, 1928; Pernkopf, 1928). They describe a ‘colic angle’ that depends for its angular shape on a ‘retention band’ in the dorsal mesentery near the stems of the primary loop and the SMA. Both structures are temporary and vanish before hernial return, but after the boundaries of the colic segments are established: the descending and ‘less than the left half of the transverse colon’ derive from the ‘median abdominal colon’, whereas the ‘remainder of the transverse’ and the ascending colon come from the distal limb of the (primary) loop (Frazer & Robbins, 1915; Hunter, 1928; Pernkopf, 1928). They also report that the first secondary loop and the ‘abdominal colon’, which make up the stems of the primary loop, do not herniate.

There are several issues with this model, the most conspicuous of which is that the midgut–hindgut junction is not associated with any histological or topographical landmark apart from the vascular arcade. Additional issues of the vascular hypothesis are that the coeliac trunk only perfuses the abdominal part of the caudal foregut, even though the entire oesophagus initially has a dorsal mesentery that is continuous with that of the caudal foregut below the diaphragm (Hikspoors et al., 2019). Similarly, the published topographic relation between the hindgut and the IMA is not robust. Pernkopf showed in a reconstruction of a 5‐week‐old human embryo (figure 41, scheme I in Pernkopf, 1928) that the roots of the SMA and IMA course towards the gut near the upper and lower boundaries, respectively, of the midgut and its mesentery, but then concluded that the midgut‐to‐hindgut boundary colocalizes with the splenic flexure after ~6 weeks of development (page 67 and figures 41, schemes II–IV; Pernkopf, 1928). We will show that, instead, the splenic flexure only gradually acquires its characteristic position and acute flexure, and that this flexure can serve as a landmark after the 10th week only. Douard et al. (2006) produced a semi‐schematic drawing of a 6.5‐week‐old human embryo (their figure 2), in which the gut resembles Pernkopf's reconstruction of the 6‐week‐old embryo strikingly, but in which the arterial vessels course through the centre of the mesenteries of the midgut and hindgut, respectively. Unfortunately, we are unable to establish the reasons why Frazer and Robbins' model has prevailed.

**FIGURE 1:**
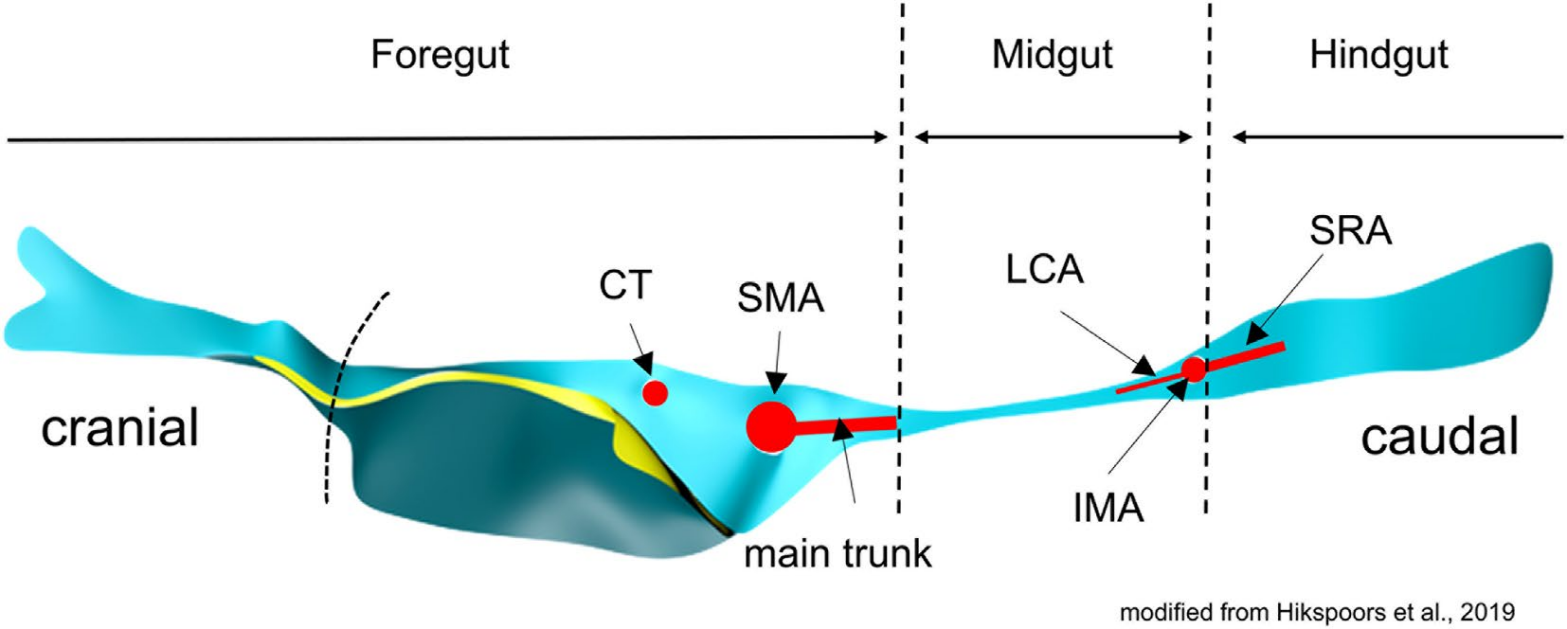
The thin mesentery locates between the trunks of the SMA and the IMA in a CS15 (∼5 weeks) embryo. The root of the dorsal mesentery, slightly modified from Hikspoors et al. (2019), is represented by a light blue horizontal strip that reflects its width. The curved dashed line indicates the developing diaphragm. The most cranial part of the dorsal mesentery is relatively thick and attached to the oesophagus. The dorsal mesentery of the caudal foregut consists of a thick mesenteric block that attaches to the liver (turquoise), lesser sac (yellow) and the mesenteries of the stomach and duodenum (light blue). The dorsal mesentery of the midgut is, by contrast, remarkably thin. The roots of the SMA and IMA occupy the cranial and caudal junctional areas surrounding the midgut mesentery, respectively. CS, Carnegie stage; CT, celiac trunk; IMA, inferior mesenteric artery; LCA, left colic artery; SMA, superior mesenteric artery; SRA, superior rectal artery.

When we studied the appearance and subsequent development of the human midgut between 5 and 12 weeks of development, we observed that the midgut was characterized by its association with a dorsal mesentery that was >3‐fold thinner than that of the adjacent mesenteries of the caudal foregut and hindgut (Figure 1; Soffers et al., 2015). This finding confirmed a 55‐year‐old, but hardly cited study (Yokoh, 1970). Furthermore, Frazer and Robbins stated in their 110‐year‐old text and a schematic drawing that the mesentery of the hindgut was thicker than that of the midgut (Frazer & Robbins, 1915; Hunter, 1928; Pernkopf, 1928). Yokoh nor Frazer and Robbins established a relation with the midgut‐hindgut junction. Based on their and our earlier observations, we hypothesized that the topographic position of the narrow‐wide transition of the dorsal mesentery persists into foetal stages of development. We then established that, at 10 weeks of development, the caudal part of the gut had sufficiently differentiated to allow the thin‐thick mesenterial boundary to be associated with the rectosigmoid junction. In addition, the trunk of the IMA proved to be independent, developmentally stable and a reliable pointer to this junction. The adhesion and subsequent fusion of the proximal part of the colic mesentery to the ventral wall of the duodenum, stomach and dorsal pancreas fixed the topographic position of the ascending limb of the colon immediately after its return from the umbilical hernia. Finally, we observed that the differentiation of the midgut and hindgut, and hence the colon and rectum, differ fundamentally in that the rectum exhibits a caudal‐to‐cranial direction of differentiation.

## 2 MATERIALS AND METHODS

### 2.1 Embryos and foetuses

The study used digitized images of serial sections of anonymized specimens from the historical collections of human embryos and foetuses of the Departments of Anatomy and Embryology, Leiden University Medical Centre (LUMC), Leiden; Amsterdam Medical Centers (AMC), Amsterdam; Radboud University, Nijmegen, The Netherlands; Georg‐August‐Universität Göttingen, Germany (Blechschmidt Collection) and Ruhr University, Bochum, Germany (Hinrichsen collection). These historical collections are exempt from ethical approval according to the Dutch regulations for the proper use of human tissue for (bio‐) medical research purposes. In addition, embryos of the Carnegie Collection in Washington DC, which are freely accessible on the internet, were included. The criteria of O'Rahilly and Müller, as modified in 2010 (O'Rahilly & Müller, 2010), were used to determine Carnegie Stages (CS) and ages of development. All specimens used are shown in Table 1. The age of the foetuses (>8 weeks of development) was based on the comparison of their crown‐rump length with that in reference autopsy studies, as described (Hülsman et al., 2024a). The standard deviation of the estimated age in autopsies was ~1 week in the second trimester.

**TABLE 1:** Overview of all used human embryos and foetuses.

Stage	Days	Number	Plane	Source	Sex
CS14‐early	33	S2201	Transverse	AMC	/
CS14‐intermediate	34	S168	Transverse	LUMC	/
CS14‐late^a^	35	S6502	Transverse	DREM	/
CS14‐late	35	1961‐06‐13	Transverse	Göttingen	/
CS15	36	1945‐10‐26	Transverse	Göttingen	/
CS15	36	S79	Transverse	LUMC	/
CS15^a^	36	S2213	Transverse	AMC	/
CS15^a^	36	S721	Transverse	DREM	/
CS16	39	S6517	Transverse	DREM	/
CS16	39	S39	Transverse	LUMC	/
CS17^a^	41	S6520	Transverse	DREM	/
CS18‐early	43	S97	Transverse	LUMC	/
CS18‐late^a^	45	S4430	Transverse	DREM	/
CS20^a^	48	S2025	Transverse	AMC	/
CS20	48	S34	Transverse	LUMC	/
CS21^a^	51	S4090	Transverse	DREM	/
CS22^a^	53	S983	Transverse	DREM	Male
CS23	56	S9226	Transverse	DREM	Male
CS23^a^	56	S48	Transverse	LUMC	Male
CS23	56	S4141	Transverse	AMC	Female
CS23	56	eyo295	Transverse	Bochum	Female
9 weeks^a^	63	S89	Transverse	LUMC	Female
9.5 weeks^a^, ^b^	67	S57	Transverse	LUMC	Female
9.5 weeks	67	S105	Transverse	LUMC	Male
10 weeks	70	S1507	Sagittal	AMC	Male
10 weeks^a^	70	S4908	Transverse	AMC	Female
11 weeks^a^	77	S1744	Transverse	LUMC	Female
11 weeks	77	S1743	Sagittal	LUMC	Female
11 weeks	77	S2410	Sagittal	LUMC	Male
11 weeks	77	S2409	Transverse	LUMC	Male
12 weeks	84	S2391	Sagittal	LUMC	Male
12 weeks^a^	84	S3291	Transverse	AMC	Male
13 weeks	91	S1742	Transverse	LUMC	Male
13 weeks	91	ME29534	Frontal	Bochum	Female
13 weeks	91	S2212	Sagittal	LUMC	Female
13 weeks^a^	91	S2383	Transverse	LUMC	Female

*Note*: Crown‐rump length of embryos was converted to days post fertilization based on O'Rahilly and Müller (2010) and weeks post fertilization based on Hülsman et al. (2024a). Staging followed the DREM classification (https://virtualhumanembryo.lsuhsc.edu), which, in turn, is based on O'Rahilly et al. (1987). The staging of the other embryos was accomplished by comparing these embryos to the DREM specimens and taking more or less advanced morphological features into account.

^a^ Reconstructed 3D‐PDF model (Cinema4D).

^b^ 9‐week‐old foetuses still have a herniated midgut, whereas the midgut hernia has resolved in 9.5‐week‐old foetuses (Blaas & Eik‐Nes, 2008).

### 2.2 Image acquisition, 3D reconstruction and visualization

Serial sections of human embryos from the AMC, LUMC and RadboudMC collections were digitized with an Olympus BX51 or BX61 microscope and the DOTSLIDE program (Olympus, Zoeterwoude, The Netherlands). The Göttingen and Bochum specimens were scanned with a Zeiss Axioscan Z1 (Carl Zeiss Microscopy, Jena, Germany). Digital images of serial sections from the Carnegie collection were obtained via the Virtual Human Embryo Project (https://virtualhumanembryo.lsuhsc.edu). All images were converted into grey‐scale ‘JPEG’ format and loaded into Amira (version 2021.2; base package; FEI Visualization Sciences Group Europe, Merignac Cedex, France). The grey‐scale images were aligned automatically with the least‐squares alignment mode, and then manually adjusted for the correct curvature of the embryonic body axis with the help of photographs and magnetic resonance images of intact human embryos of the same age (Pooh et al., 2011). Structures of interest were segmented manually and reconstructed three‐dimensionally with the Amira program. Small deformations of individual sections due to the histological processing and stepwise stacking of sections confer distracting noise on the 3D reconstructions. Therefore, polygon meshes from all reconstructed materials were exported via ‘vrml export’ to Cinema4D (MAXON Computer GmbH, Friedrichsdorf, Germany) and remodelled using the Amira model as a template. The accuracy of the remodelling process was safeguarded by simultaneous visualization in Cinema4D of the original output from Amira and the remodelled Cinema model. The Cinema4D models were transferred via ‘wrl export’ to Adobe Acrobat version 9 (https://www.adobe.com) to generate interactive 3D Portable Device Format (PDF) files, which is an easily accessible format for 3D visualization.

### 2.3 Measurements and assessments

Volume measurements were performed with the statistical analysis mode of Amira. The length or width of structures was measured with the spline option in Cinema4D.

### 2.4 Orientation and assessments

The body axis is defined as the axis passing through the vertebral column without the sacrum and coccyx. The terms cranial and caudal are used to identify changes along the body axis, and dorsal and ventral are used to identify changes perpendicular to the body axis.

### 2.5 Terminology

We recognize proximal and distal limbs of the definitive midgut based on their position relative to the vitelline duct. The distal limb of the midgut encompasses the distal part of the ileum and the entire colon, if the midgut‐hindgut junction is accepted to colocalize with the rectosigmoid junction. In foetuses, we recognize ascending or presplenic and descending or post‐splenic limbs of the colon. This topography becomes established immediately after the hernial return and is present in all mammalian foetuses. The ascending or presplenic limb becomes subdivided into the ascending and transverse colons as soon as a hepatic flexure can be recognized. The descending or postsplenic limb becomes divided into the descending and sigmoid colons as soon as the descending colon becomes a secondarily retroperitoneal structure. The degree of mesenteric fusion of the post‐splenic colon with the dorsal body wall is variable.

Three‐dimensional reconstructions presented in PDF format.

In addition to the Figures, the evidence for our descriptions can be inspected in the corresponding interactive 3D‐PDFs (Figures [Link], [Link]). The reader is, therefore, encouraged to read the text and inspect the corresponding interactive PDFs simultaneously. This is because the rotational options of the 3D‐PDFs (‘live’ images) allow for a much better understanding of the complex local topography than do ‘still’ images and text. The 3D‐PDFs can also be used to identify or verify the identity of a structure in a figure: after opening and positioning the reconstruction as seen in the figure, marking or unmarking a structure in the model tree will link the image to the corresponding name (Table 2).

**TABLE 2:** List of abbreviations and colour codes used in figures.

C	Colon	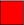	Artery
Cau	Caudal	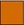	Allantois
Cr	Cranial	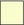	Bone
CRL	Crown‐rump length	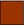	Cloaca
CS	Carnegie stage	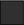	Caudal foregut
CT	Celiac trunk	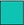	Duodenum
D	Dorsal	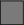	Foregut mesentery
D	Duodenum	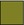	Caudal foregut mesenchyme
DP	Dorsal pancreas	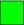	Hindgut
HG	Hindgut	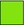	Hindgut mesenchyme
IMA	Inferior mesenteric artery	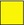	Hindgut mesentery
L	Left	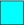	Insertion of mesentery into dorsal abdominal wall
L4	Fourth lumbar vertebra	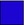	Midgut
L5	Fifth lumbar vertebra	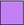	Midgut mesenchyme
LCA	Left colic artery	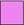	Midgut mesentery
J	Jejunum	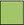	Oesophagus
M	Mesentery	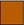	Pancreas
MCA	Middle colic artery	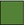	Pancreatic duct
MG	Midgut	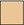	Stomach
R	Rectum	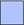	Caecum and appendix
R	Right		
S	Stomach		
SA	Sigmoid arteries		
SMA	Superior mesenteric artery		
SRA	Superior rectal arteries		
V	Ventral		
VD	Vitelline duct		
VP	Ventral pancreas		

## 3 RESULTS

### 3.1 The early development of the colon

**FIGURE 2:**
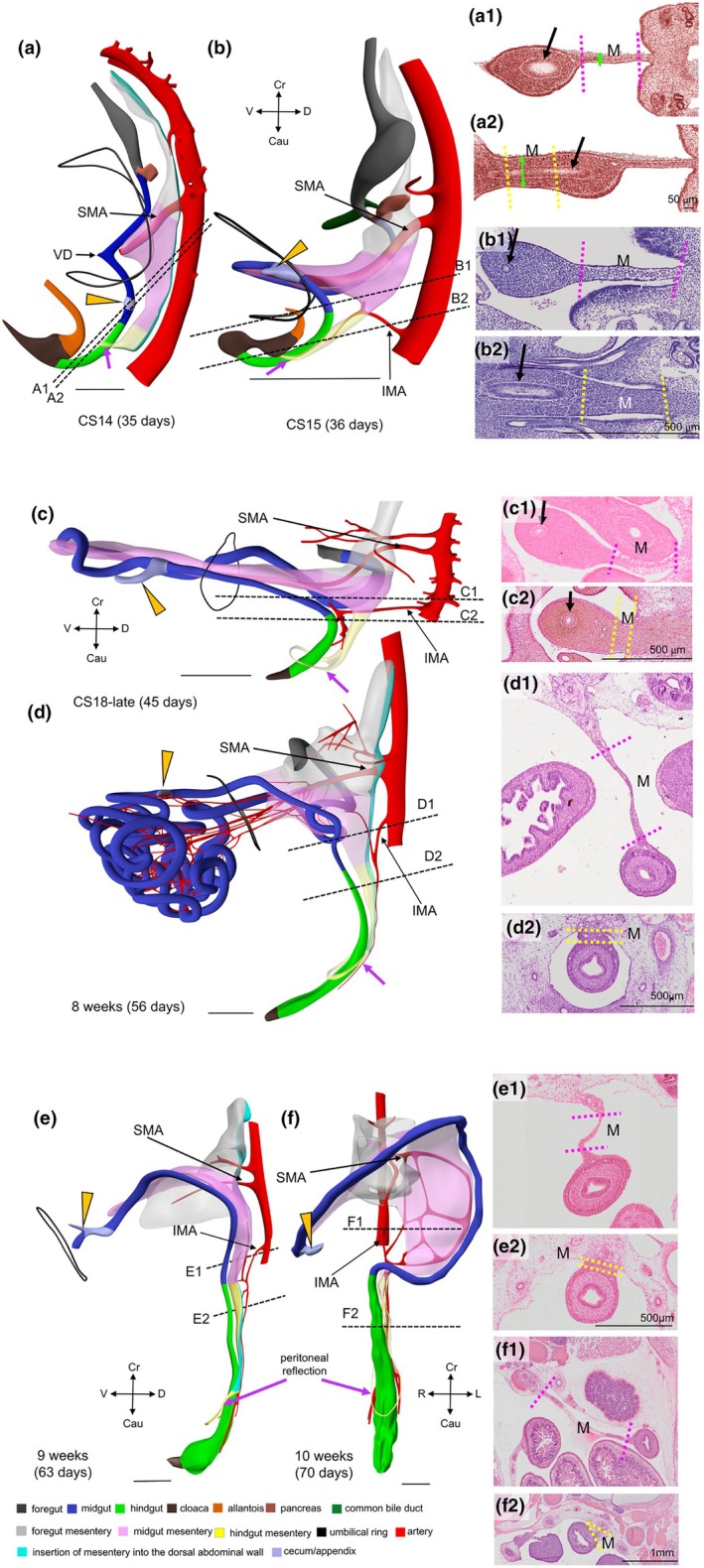
A thin dorsal mesentery identifies the midgut. 3D models (a–e) show left‐sided views and model f a ventral view of the midgut and hindgut at CS14‐early, CS15, CS18‐late, CS23 (8 weeks), 9 and 10 weeks. The dashed black lines in panels (a–f) indicate the position of the sections. The caudal foregut, midgut and hindgut are colour‐coded grey, blue and green, respectively, in panels (a–f), whereas thin and thick mesenteries are delimited by dashed purple and yellow lines, respectively, in panels (a1–f1 and a2–f2). The orange arrowheads indicate the caecum, and the black contours in panels (a–e) the umbilical ring. The caecum returns into the abdominal cavity at 9.5 weeks, as shown by the yellow arrowhead at the foetal side of the umbilical ring (panel e). The thick black arrows in the sections of panels a1, a2–c1, c2 point at the location of the gut epithelium. The mesenteric thickness was determined perpendicular to the mesenteric sheets in histological sections (panels a1–f1 and a2–f2). The topographic position of the thin‐thick mesenterial junction (panels e and f) positions the midgut–hindgut junction of the embryo at the rectosigmoid junction. The topographic position of the first branching nodes of the trunks of the SMA and IMA (panels a–f) corresponds with the cranial and caudal margins, respectively, of the thin mesentery of the midgut. The thick yellow ring surrounding the rectum (purple arrows in panels a–f) represents the peritoneal reflection. CS, Carnegie stage; IMA, inferior mesenteric artery; M, mesentery; SMA, superior mesenteric artery. Bar (a–f and a'–f') = 1 mm.

**FIGURE 3:**
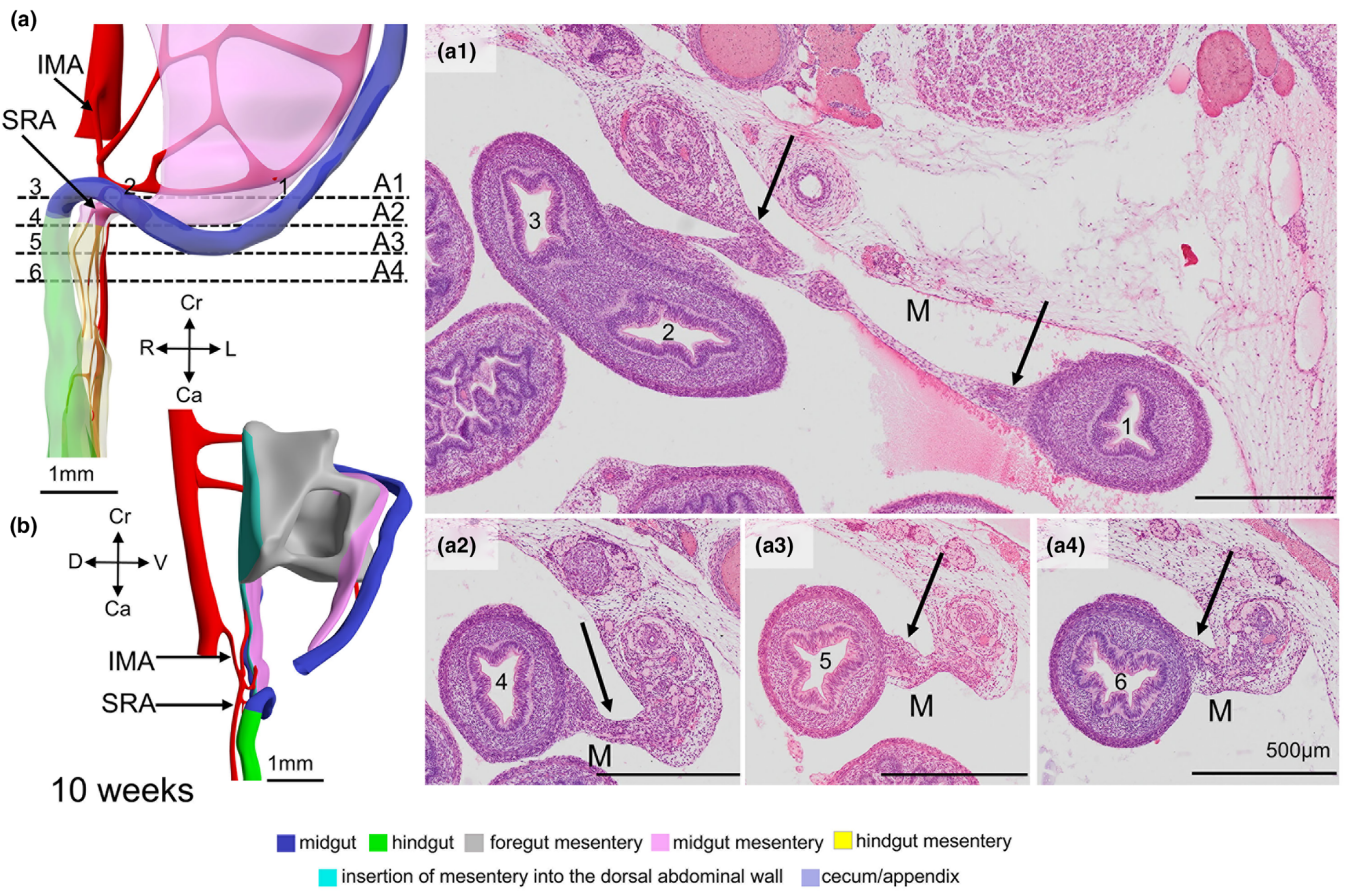
The histology of the rectosigmoid junction at 10 weeks. Ventral (a) and right lateral (b) views of the rectosigmoid junction at 10 weeks (same embryo as shown in Figure 2f). Dashed black lines in panel (a) indicate the level of sections shown in panels a1–a4. The numbers in gut lumens (1–6) of panels a1–a4 correspond to that in panel (a), with loops 1–3 representing the near‐horizontal part of the sigmoid colon and loops 4–6 representing the near‐vertical part of the upper rectum. Black arrows in panels a1–a4 mark the mesenteric attachment, which increase gradually in thickness between panels a2 and a4. The epithelial lining of loops 1–6 further demonstrates the progressive differentiation from colic to rectal epithelium. IMA, inferior mesenteric artery; M, mesentery; SRA, superior rectal artery.

CS 14 (~4.5 weeks of development) is the earliest stage at which we can identify the thin midgut mesentery (encoded pink in Figures 2a and S2). It is triangular in shape with the regressing vitelline duct at its ventral apex. The position of the vitelline duct divides the midgut into cranial and caudal limbs. The SMA courses towards the vitelline duct and yolk sac along the cranial limb of the midgut. At all ages, the >3‐fold difference in width of the midgut and hindgut mesenteries was used as a parameter to localize the transition between both parts of the gut (compare panels a1–f1 with panels a2–f2 of Figure 2). In CS14 embryos, the wide caecum (orange arrows) has a thin mesentery only (Figure 2a). The caudal boundary between the thin and thick parts of the dorsal mesentery immediately follows the caecum, which suggests that the colon is still short at this stage, if it initially exists at all (Figures 2a and S2). The CS15 embryos (~5 weeks of development; Figures 2b and S3) also demarcate the topography of the thin dorsal mesentery of the midgut well: the cranial part begins to loop into the umbilical hernia and is dominated by the large SMA that accompanies it, whereas the caudal, colic part follows a straight course in dorsocaudal direction. Comparison of Figure 2a–c shows that the length of the colon (caecum (grey‐blue) to hindgut (green)) increases rapidly between CS14 and CS18. The IMA has formed and reaches the gut via the short transitional zone between the thin and thick mesentery (Figure 1). The consistent course of its ‘trunk’ (proximal part) is shown in Figures 2b,c and 3.

**FIGURE 4:**
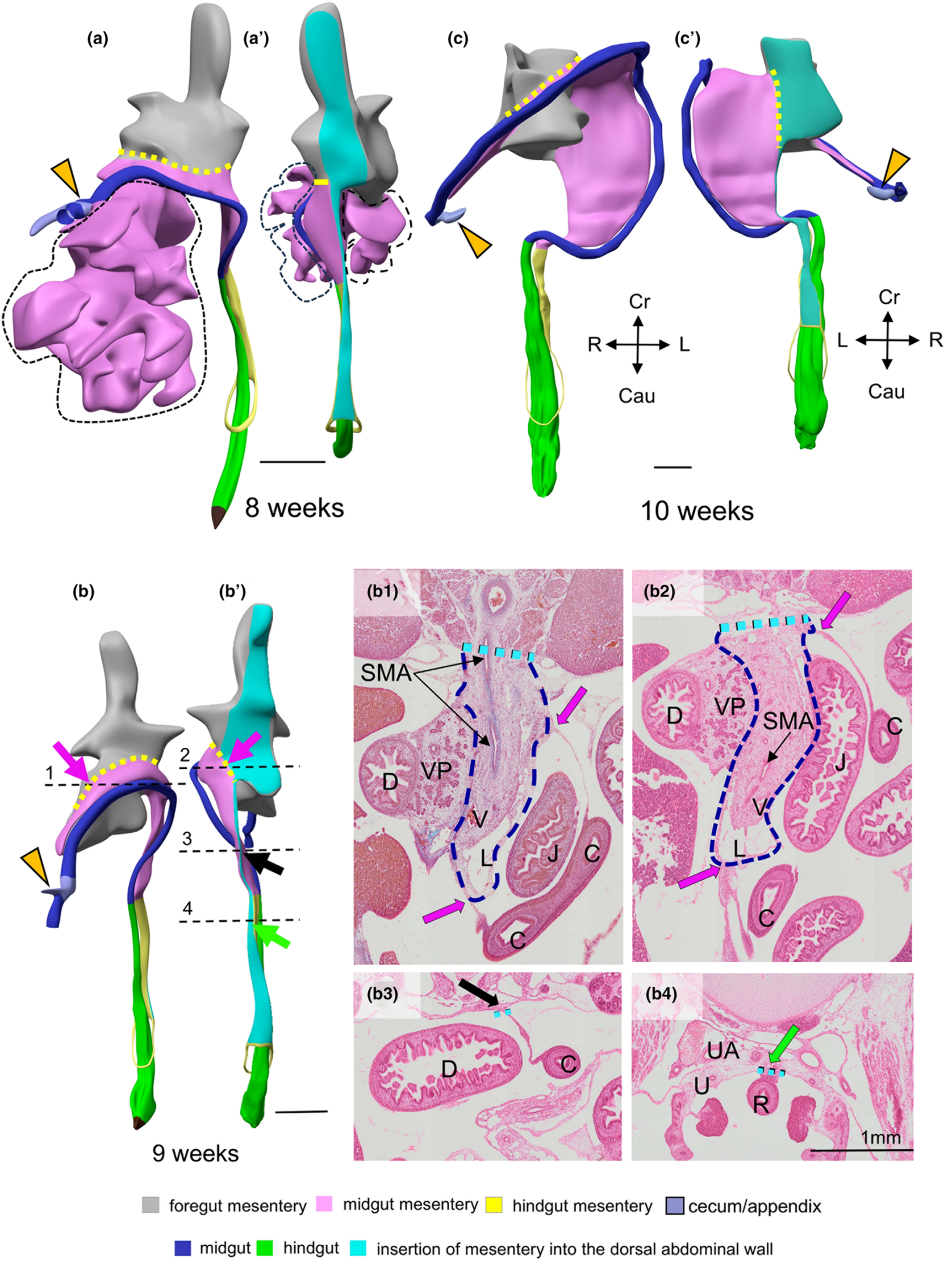
The junction between the thick caudal–foregut and thin colic mesenteries. Panels a (8 weeks), b (9 weeks) and c (10 weeks) show ventral, and panels a'–c' dorsal views of the caudal foregut (grey), midgut (pink) and hindgut (yellow) parts of the dorsal mesentery. In panel (a), the small‐intestinal epithelial tube is removed, but its mesentery (pink) is present within the dashed black contour. In panels (b) and (c), both the small‐intestinal epithelial tube and its mesentery are removed, so that the remaining pink mesentery represents the mesocolon only. The orange arrowheads indicate the caecum (grey‐blue). The position of sections b1–b4 is indicated by the black dashed lines in panels (b) and (b'). The cyan areas in panels (a'–c'), and the cyan dashed lines in sections b1–b4 indicate the dorsal attachments of the mesenteries of the caudal foregut (panels b1 and b2) and the colon (panels b1–b3). Sections b1 and b2 show that, at 9 weeks, the ascending limb of the thin colic mesentery attaches to the left side of the trunk of the thick dorsal mesentery of the caudal foregut (bright purple arrows), whereas the descending thin limb attaches directly to the midline of the dorsal abdominal wall (Section b3; black arrow). The bright green arrows in panels b' and b4 identify the attachment of the thick rectal mesentery on the dorsal body wall. The yellow dashed lines in panels (a–c) and (a'–c') and the purple arrows in sections b1 and b2 present the 3D distribution of the attachment of the thin mesentery of the ascending limb of the colon (pink) to the trunk of the thick mesentery of the caudal foregut (grey). C colon; D, duodenum; J, jejunum; L, lymphatic vessels; R, rectum; SMA, superior mesenteric artery; U, ureter; UA, umbilical artery; V, veins; VP, ventral pancreas. Bar = 1 mm.

**FIGURE 5:**
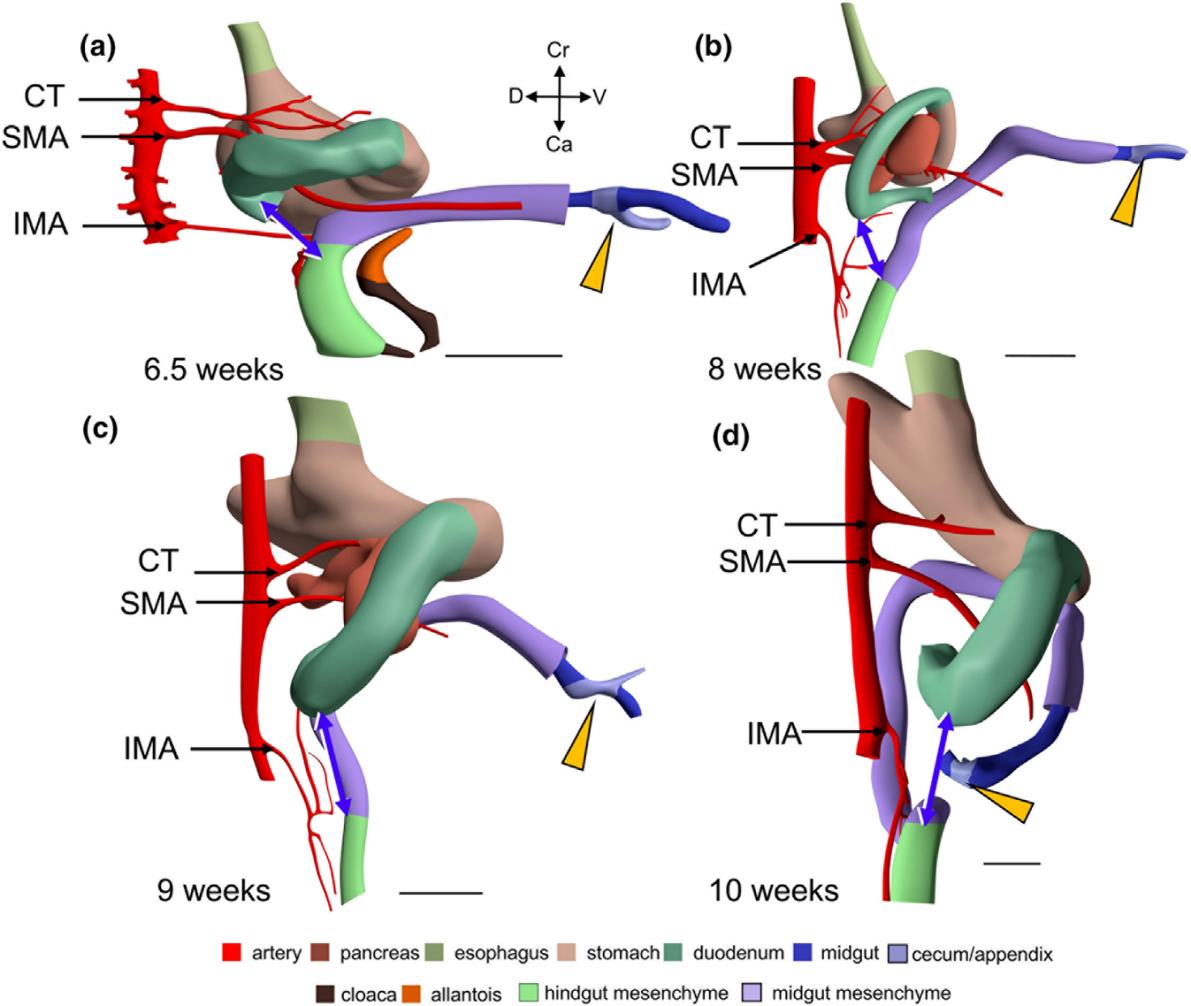
The horizontal part of the duodenum and the rectosigmoid junction remain closely adjacent during midgut herniation and its return. Rightward views of the wall of the duodenum and the rectosigmoid junction at 6.5 (a), 8 (b), 9.5‐early (c) and 10 weeks (d) of development. The rectosigmoid junction connects the midgut (purple) and hindgut (light green). The abdominal aorta and its ventral branches are depicted in red. The middle part of the pancreas (orange‐brown, only shown in panels b and c) lies in between the CT and SMA. The orange arrowheads indicate the caecum (grey‐blue). The double‐headed blue arrows in all four panels indicate the distance between the horizontal (third) part of the duodenum (start of primary loop of the midgut) and the rectosigmoid junction (end of primary loop). Despite the pronounced changes in orientation of the arteries, especially that of the trunk of the IMA (see also Figure 8), the horizontal part of the duodenum stays close to the rectosigmoid junction. This finding shows that the root of the primary intestinal loop retains its configuration and topography during midgut herniation and its return. CT, celiac trunk; IMA, inferior mesenteric artery; SMA, superior mesenteric artery. Bar = 1 mm.

The difference in the topography of the small intestine, colon and their respective mesenteries during stages CS15–18 is striking (Figures 2, S3 and S4). In agreement with the course of the primary loop, the colon and its mesentery are predominantly present in the upper and left parts of the abdomen and hernial sac, whereas the small intestine and its mesentery are mainly present in the right and lower parts of the abdomen and hernial sac (Figures 2, 4 and S3–, S8). The thick mesentery of the caudal foregut, therefore, ends where the primary loop develops, which is where the distal duodenum and colon are closest between 5 and 10 weeks of development (double‐headed blue arrows in Figure 5a–d).

### 3.2 The midgut‐to‐hindgut transition is located at the rectosigmoid junction

**FIGURE 6:**
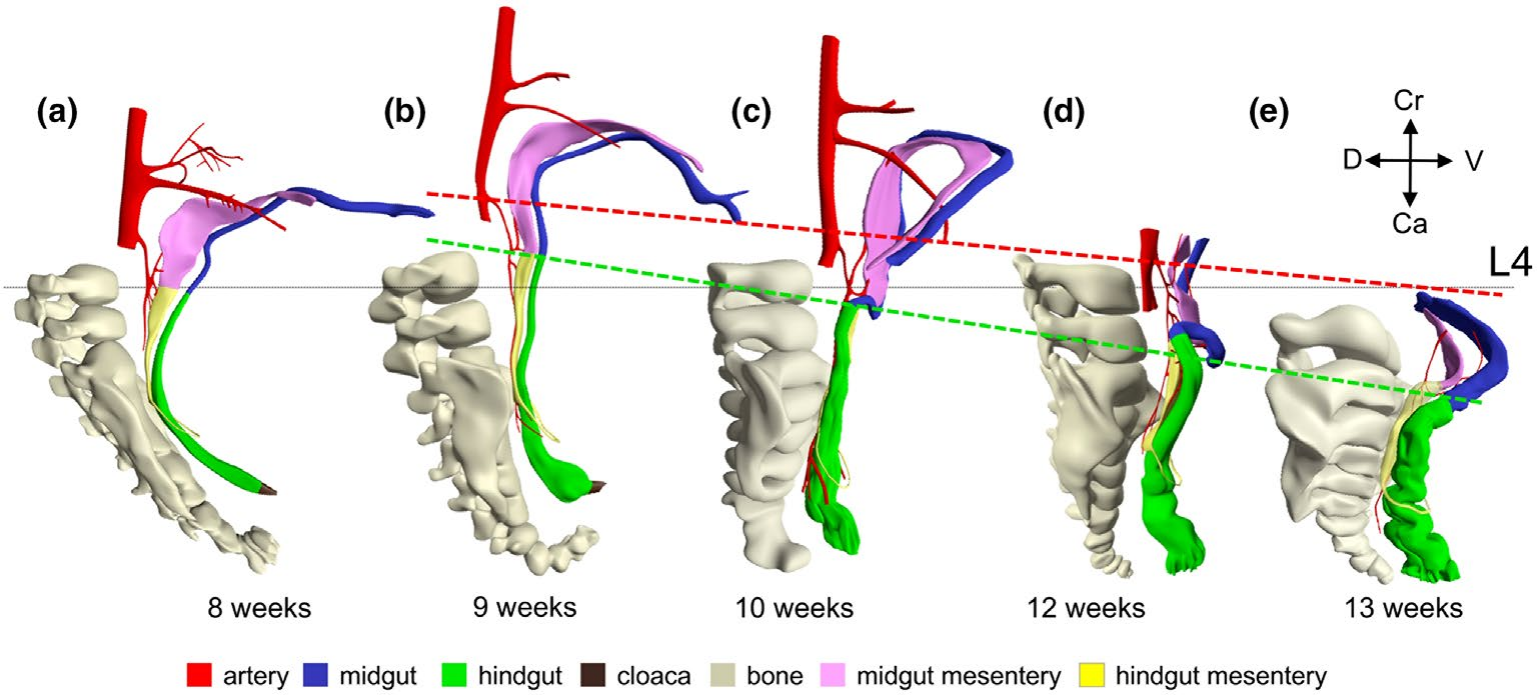
The descent of the rectosigmoid junction. The panels show a rightward view of the vertebrae, ventral abdominal arteries and the thin and thick mesenteries of the colon and rectum at 8 (a), 9 (b), 10 (c), 12 (d) and 13 weeks (e). The thin black line represents the position of lumbar vertebra 4. The bony structures in all reconstructions are comparable in size. The green dashed line shows the descent of the rectosigmoid junction from L3‐4 at 9 weeks to S1 at 13 weeks. The red dashed lines show the position of the root of the IMA. The root of the IMA descends slightly slower than the rectosigmoid junction due to a slower growth of the abdominal aorta. L, lumbar vertebrae.

Following the transition of a thin to a thick mesentery in the caudal part of the gut between 4.5 (CS14) and 10 weeks, we could map this transition topographically and histologically in the 10th week of development to the junction between the sigmoid colon and the rectum (Figure 2f). Herniation of the midgut into the umbilical coelom occurs in the 6th week (Figures 2b,c, S3 and S4), while the hernial return of the midgut takes place in the 10th week (Figures 2f and S8). The identification of the rectosigmoid bend as the junction between the mid‐ and hindgut can, therefore, only be made shortly after the midgut has returned from the umbilical hernia. The rectosigmoid bend gradually becomes more pronounced, while the position of the rectosigmoid junction descends from vertebra L4 at 10 weeks to vertebra S1 at 13 weeks (Figure 6). During the herniation period, the colon courses dorsally in a left paramedian plane from the upper‐left position in the hernial sac towards the stomach and then caudally without any sharp bend (Figure 2b–e). In the 9th week, the splenic bend becomes sufficiently pronounced to distinguish the ascending and descending limbs of the colon, but the junction between thin (midgut) and thick mesentery (hindgut) is not yet marked by an acute bend, or other marker (Figures 2e and S7). During hernial return, the ascending part of the colon (with a thin mesentery) moves from the upper‐left part of the hernial sac to a location just cranial to the right iliac crest (better known as McBurney's point). The course of the ascending limb of the colon, therefore, changes from one in a sagittal plane during herniation to one in a frontal plane after hernial return. Meanwhile, the descending limb of the colon establishes its typical left‐sided position in the abdominal cavity (Figures 2f and S8), so that its course must turn medially and then caudally to reach the pelvic peritoneal cavity. The resulting acute rectosigmoid bend also displays the transition from a thin to a thick mesentery, that is, co‐localizes with the midgut–hindgut junction. In addition, it exhibits the characteristic topography (located at the entrance to the lesser pelvis) and shape (bend from left to caudal) of the rectosigmoid junction.

### 3.3 Nerves and vessels thicken the mesentery locally

**FIGURE 7:**
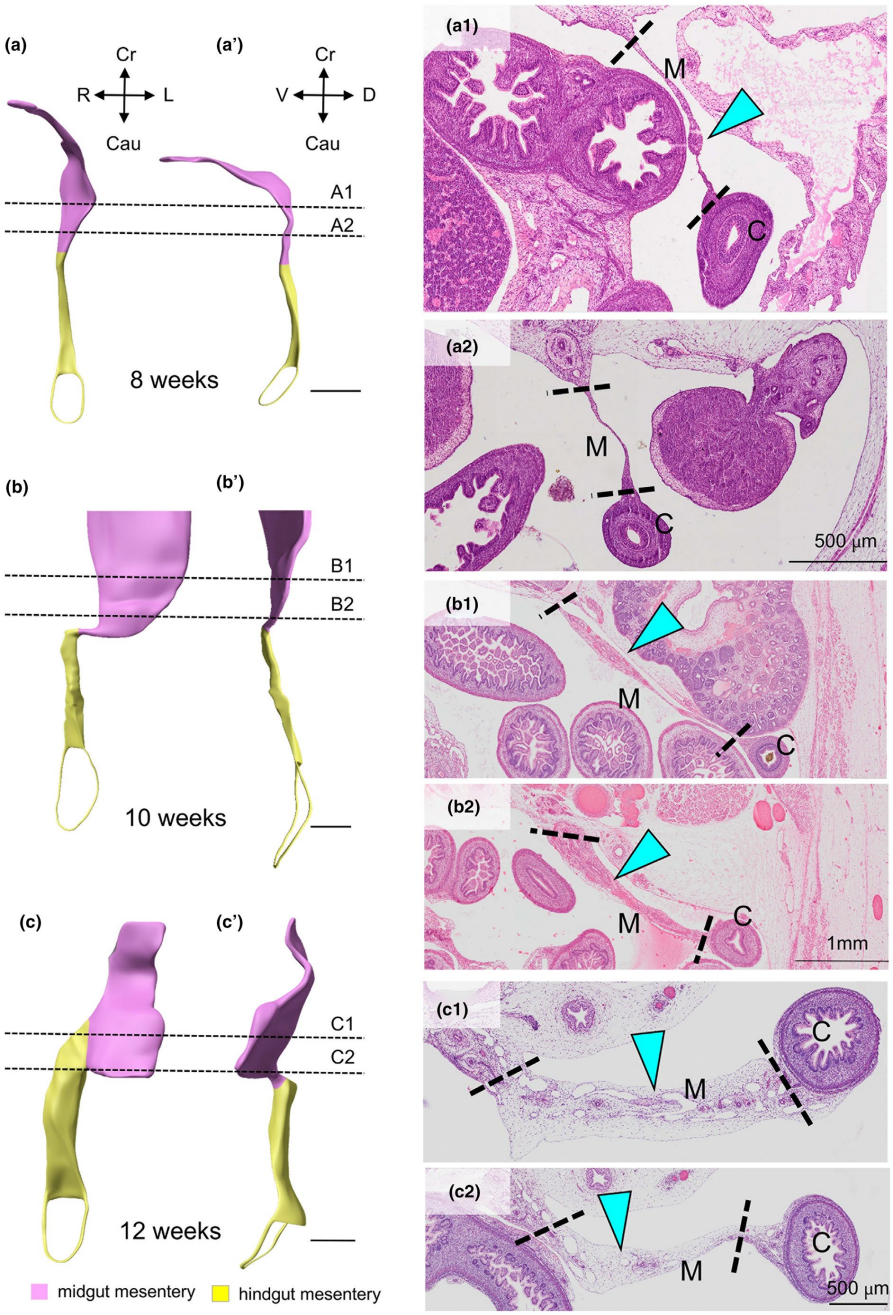
Structural changes in the thin dorsal mesentery of 8–12‐week foetuses. Ventral (panels a–c) and left‐sided views (panels a'–c') of the thin (pink) and thick (yellow) dorsal mesenteries. The histological sections in subpanels 1 and 2 of panels a–c are located at the levels indicated by the dashed lines in panels a–c and a'–c'. Mesenteries are sectioned perpendicularly to their surface. The figure shows that the mesenteries still have a ‘thin identity’ (panel a2) but have locally become thicker due to the ingrowth of vessels and nerves (cyan arrowheads in panels a1, b1, b2, c1 and c2). C, colon; M, mesentery. Bar = 1 mm.

To reach the conclusion that the midgut–hindgut junction corresponds with the rectosigmoid junction, we excluded tissue masses in the mesentery that could be assigned to the ingrowth or expansion of blood and lymph vessels and of nerves. These swellings become a prominent feature during and after the 8th week of development, but can be distinguished from genuinely thick mesentery, because they are always flanked by thin mesentery. Figure 7 shows examples of such swellings in the mesentery of the midgut at 8, 10 and 12 weeks of development. The mesentery of these 8‐ to 12‐week‐old foetuses, with the sections spaced ~1 mm apart, shows that dilated erythrocyte‐free vessels increase the width of the small‐intestinal mesentery locally (cyan arrowheads in the histological sections shown in Figure 7). Because of the absence of blood cells, the vessels are probably lymph vessels that have become engorged preceding the death of the foetus (Hikspoors et al., 2022).

In summary, we could localize the junction between the midgut and hindgut at the rectosigmoid junction after 10 weeks of development, using the change in thickness of the dorsal mesentery and the sharp bend in the course of the gut at its entrance into the lesser pelvis as criteria. Although the width of the mesentery is a simple and reliable parameter to identify the midgut and hindgut boundary, we looked for additional landmarks to corroborate our conclusion.

### 3.4 The course of the trunk of the IMA identifies the boundary between thin and thick mesentery

**FIGURE 8:**
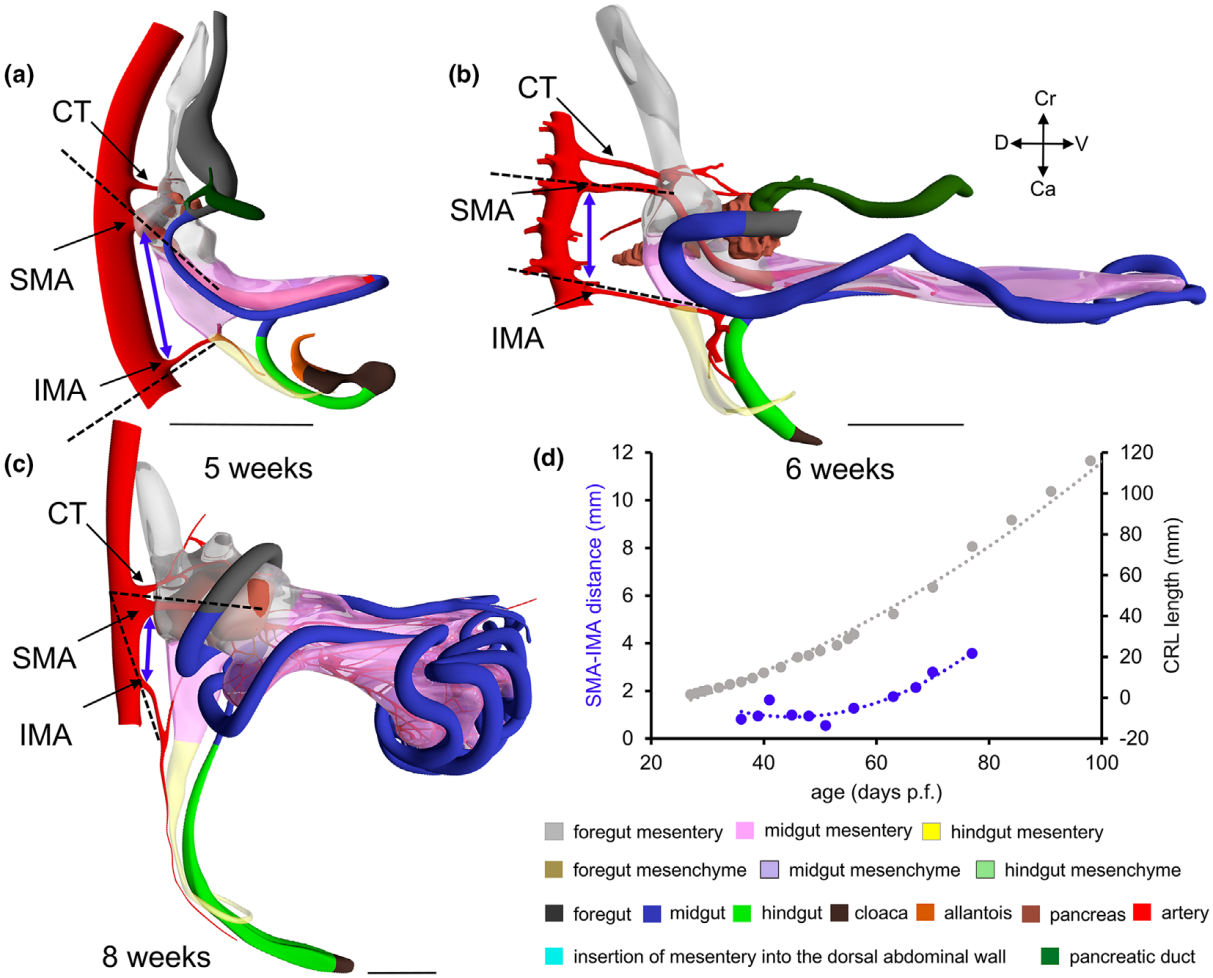
The thin mesentery of the midgut is localized between the SMA and IMA. Panels (a–c) display the mesentery of the midgut (pink) anchored between the trunks of the SMA and IMA (black dashed lines). Panel (d) shows the developmental changes in the distance between the roots of the SMA and IMA (double‐headed blue arrows; *R* ^2^ = 0.91). The distance between the roots of the SMA and IMA does not change between 5 and 8 weeks, but the angle between the course of the trunks of the SMA and IMA (black dashed lines in panels a–c) increases. Starting at 9 weeks, however, the distance between the roots of the SMA and IMA (blue dots and line in panel d) follows that of the crown‐rump length of the foetuses (grey dots and line in panel d). C, colon; CT, celiac trunk; D, duodenum; IMA, inferior mesenteric artery; J, jejunum; R, rectum; SMA, superior mesenteric artery. Bar = 1 mm.

The course of the IMA seemed a promising second criterion to identify the midgut–hindgut junction. The IMA becomes identifiable at CS15 (Figures 2b, 8a and S3). At that stage, its root corresponds in location with the junction between the thin and thick parts of the dorsal mesentery (Figures 8a and S3). Between 6 and 8 weeks of development, the trunk of the IMA reorients itself in a caudal direction (Figure 8c). Figure 8d (blue dots) shows that the distance between the roots of the SMA and IMA does not change between 5 and 8 weeks, whereas the midgut and its adjacent part of the mesentery increase in length. This differential growth can be deduced from the change in orientation of the trunks of the SMA and IMA: in the sagittal plane, their course changes from converging at 5 weeks (Figure 8a), via parallel at 6 weeks (Figure 8b), to diverging at 8 weeks (Figure 8c). Thereafter, the distance between the roots of the SMA and the IMA (blue dots in panel 8D) increases at a similar rate as the crown‐rump length of the embryo (grey dots). These data show that the 3D orientation of the trunk of the IMA between its root and its first branching node points at and, hence, identifies the position of the rectosigmoid junction between 5 and 13 weeks of development (Figures 2b–f, 8a–d and S3–, S5).

### 3.5 The first colic arterial arcade is established after hernial return

**FIGURE 9:**
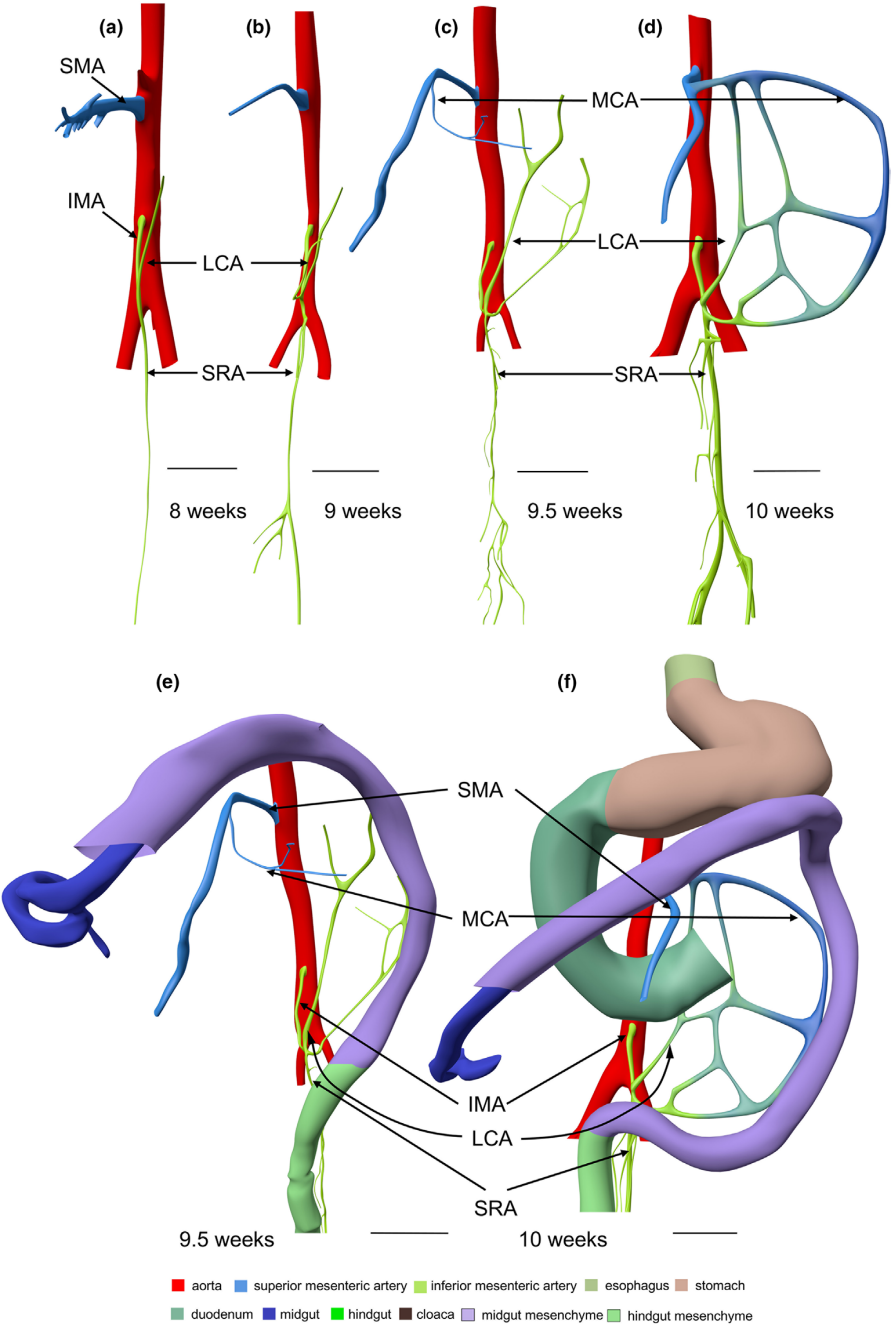
The development of the arterial arcade between the SMA and IMA. Ventral view of the development of the superior and inferior mesenteric arteries (colour‐coded blue and green, respectively) at 8 (panel a), 9 (panel b), 9.5 (panel c) and 10 weeks (panel d). The MCA (branch of SMA) and LCA (branch of IMA) form an arcade between SMA and IMA between 9.5 and 10 weeks. Note that Riolan's arc between the SMA and IMA forms, but that the marginal artery in the splenic flexure is still absent (panel f). IMA, inferior mesenteric artery; LCA, left colic artery; MCA, middle colic artery; SMA, superior mesenteric artery; SRA, superior rectal artery. Bar = 1 mm.

Figure 9 shows that the trunks of the SMA and IMA form a connecting arterial arcade, but also that this arcade only forms in the 10th week of development. The criterion of the perfusion zones of the SMA and IMA as a determinant of the midgut–hindgut junction, therefore, develops no less than 5 weeks after the thin dorsal mesentery becomes identifiable as a boundary marker for the midgut.

### 3.6 The appearance of the flexures of the developing human colon

**FIGURE 10:**
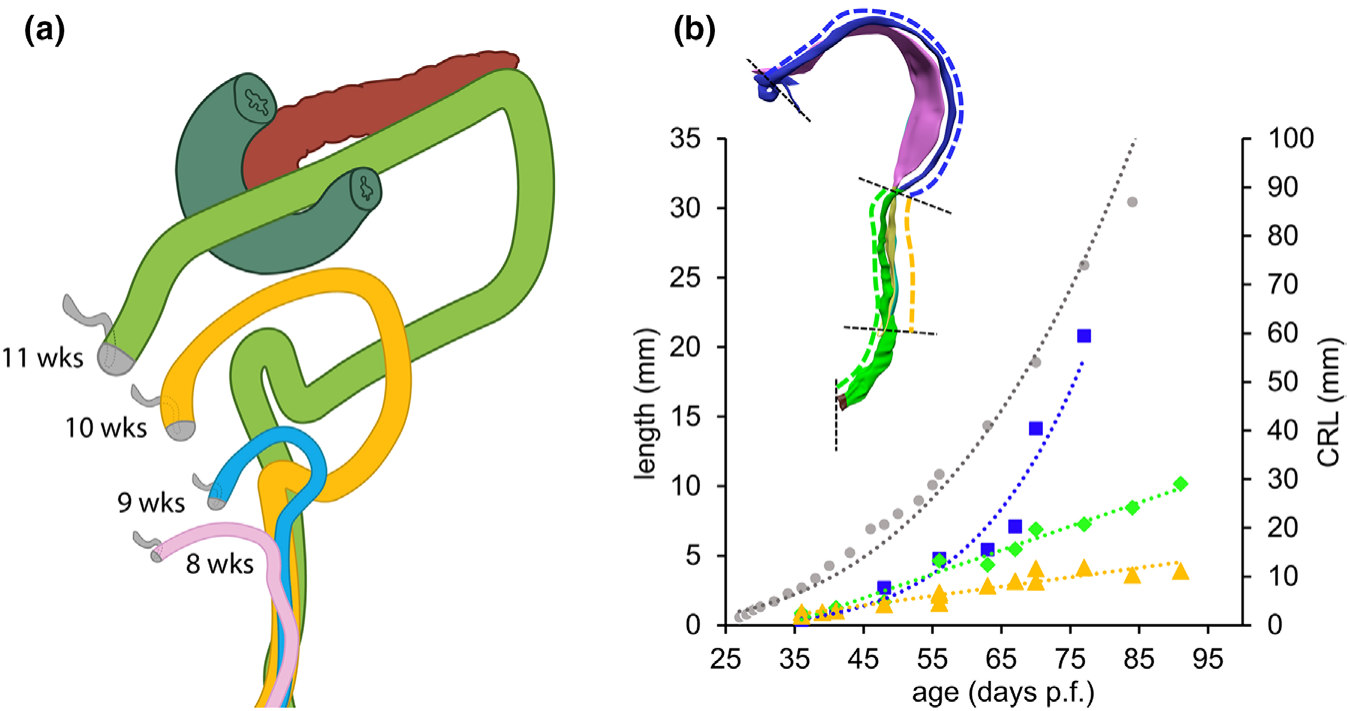
The longitudinal growth of the colon and rectum. The size and shape of the colon and hindgut/rectum at 8 (pink), 9 (light blue), 10 (orange) and 11 (green) weeks show its expansion in the abdominal cavity (panel a). The figure was modelled after figure 18 in Frazer and Robbins (1915), using our own data. The pancreas is coded by brown and the duodenum by a dark green colour. The sigmoid part of the mesocolon begins to descend to form the sigmoid loop at 10 weeks. The inset in panel (b) shows a left‐sided view of the dorsal thin (pink) and thick (yellow) parts of the dorsal mesentery of the colon (blue) and hindgut/rectum (green). Note that the distal part of the rectum has no mesentery. Panel (b) shows the outcome of the measurements. The dashed blue line (*R* ^2^ = 0.97) represents the measured length of the colon, whereas the dashed orange line (*R* ^2^ = 0.89) shows the length of the hindgut with a mesentery, whereas the dashed green line (*R* ^2^ = 0.97) shows the length of the entire hindgut. The dashed grey line represents the crown‐rump length of the embryo or foetus for reference. The graph shows that the hindgut/rectum grows linearly, with no difference between the upper part with a (thick) mesentery and the lower, retroperitoneal part. In contrast, the length of the colon increases exponentially and at a similar rate as the entire embryo or foetus in the period studied.

In human foetuses, as in most mammals, the colon forms distinct ascending and descending limbs only after the return of the umbilical hernia (compare Figures 2e, 4b and 9e with Figures 2f, 4c and 9f). Instead, the right‐sided ascending part of the colon courses directly from the caecum in the right lower abdomen to the still obtuse colic bend in the left upper abdomen (Figure 10a). The left‐sided descending colon takes the reverse course, descending from the colic bend to the rectosigmoid junction at the entrance into the pelvic peritoneal cavity. The developmental changes in the course of the colon are shown as overlays in Figure 10a. The course of the colon is similar to that in rodents until the return of the physiological hernia into the abdominal cavity at 9.5 weeks. From 10 weeks onwards, the colic bend becomes more acute and is then usually described as the ‘splenic flexure’ (Figure 10a). The rectosigmoid junction also develops a sharp bend at that time. The configuration of the colon and rectum in Figure 10a suggests that the colon grows faster than the rectum. In agreement, Figure 10b shows that growth of the colon (blue symbols) and rectum (green and yellow symbols) cannot be distinguished until ~9 weeks of development. Thereafter, the length of the colon increases exponentially, whereas that of the rectum continues to increase linearly, regardless of whether there is (green) or is not (yellow) a rectal mesentery. The downward growth of the distal portion of the colon reflects the forming of the sigmoid colon and causes the characteristic bend at the recto–sigmoid junction.

### 3.7 The topographic position of the ascending part of the foetal colon becomes fixed soon after the return of the physiological hernia

**FIGURE 11:**
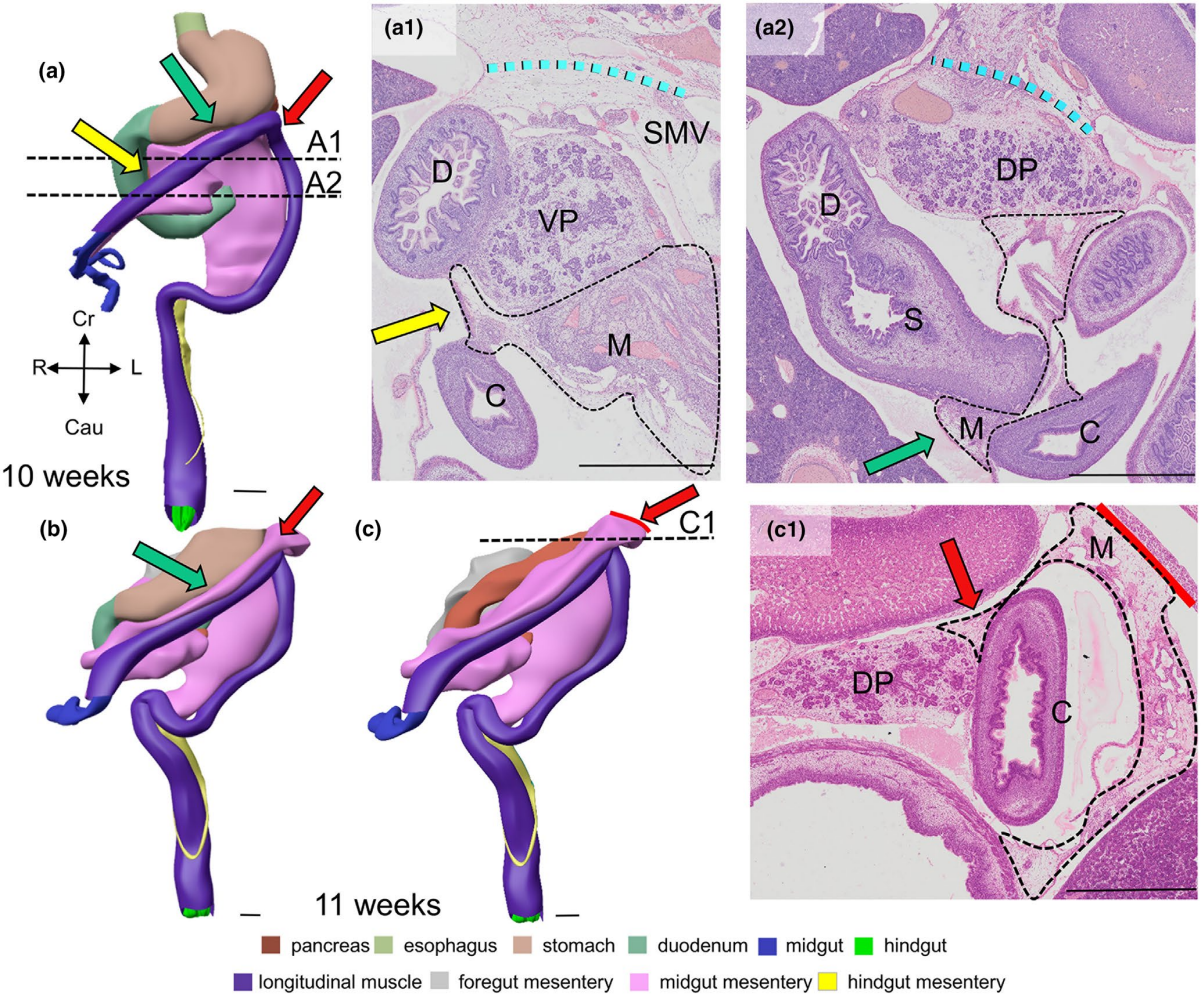
Adhesions form between the ascending part of the foetal colon and the duodenum, stomach and pancreatic tail. Ventral views of the colon at 10 (panel a) and 11 weeks (panels b and c) show the topographic relation between the colic mesentery and the duodenum, stomach and pancreas. The dashed black lines in panels (a) and (c) indicate the position of the sections (panels a1, a2, and c1). After the resolution of the physiological hernia at 9.5 weeks, the mesentery of the colon (dashed black contours in panels a1 and a2) adheres almost immediately to the ventral surface of the duodenum (panels a and a1, yellow arrows) and stomach (panels a and a2, green arrows). The cyan‐dotted lines in panels a1 and a2 indicate the attachment of the duodenum and dorsal pancreas to the dorsal abdominal wall via their dorsal mesentery. The colic bend (red arrows in panels a–c) has become a sharp splenic flexure at 10 weeks (red arrow in panel a) and adheres to the tail of the pancreas via its mesentery (dashed black contour in panel c1) at 11 weeks. Note that the stomach (colour‐coded brown in panel (b)) is not shown in panel (c) to reveal the dorsal pancreas (colour‐coded brick). C, colon; D, duodenum, DP, dorsal pancreas; M, mesentery; S, stomach; SMV, superior mesenteric vein; VP, ventral pancreas. Bars = 1 mm.

To establish more definitively that the midgut–hindgut junction colocalizes with the rectosigmoid junction, we sought to demonstrate that the position of the junctions between the ascending and descending limbs of the colon no longer changes after the return of the physiological hernia. At 10 weeks, the distal part of the ascending limb of the colic mesentery fuses with the ventral surfaces of the duodenum, ventral pancreas (yellow arrows in Figure 11a,a1) and stomach (green arrows in panels Figures 11a,a2, S8 and S9). At 11 weeks, the splenic bend has established a connection with the tail of the dorsal pancreas (red arrows in panels Figures 11b,c,c1 and S9). These findings demonstrate that the distal part of the ascending limb of the colon becomes fixed to the duodenal bend, the pancreas and the stomach at 11 weeks, that is, within ~10 days after the hernial return. The implication of this finding is that the ascending part of the foetal colon acquires a fixed position, even though the duodenum and pancreas themselves still have a thick dorsal mesentery (dashed cyan lines in Figures 4b1,b2 and 11a1,a2) that has yet to adhere and fuse with the dorsal wall of the peritoneum in 11‐week‐old human foetuses. The adhesions of the mesentery of the colon to the ventral surfaces of the upper abdominal organs should not be confused, therefore, with the fusion of the dorsal surfaces of these organs (and those of the ascending and descending colon) with the dorsal body wall.

### 3.8 The hindgut differentiates from caudal to cranial

**FIGURE 12:**
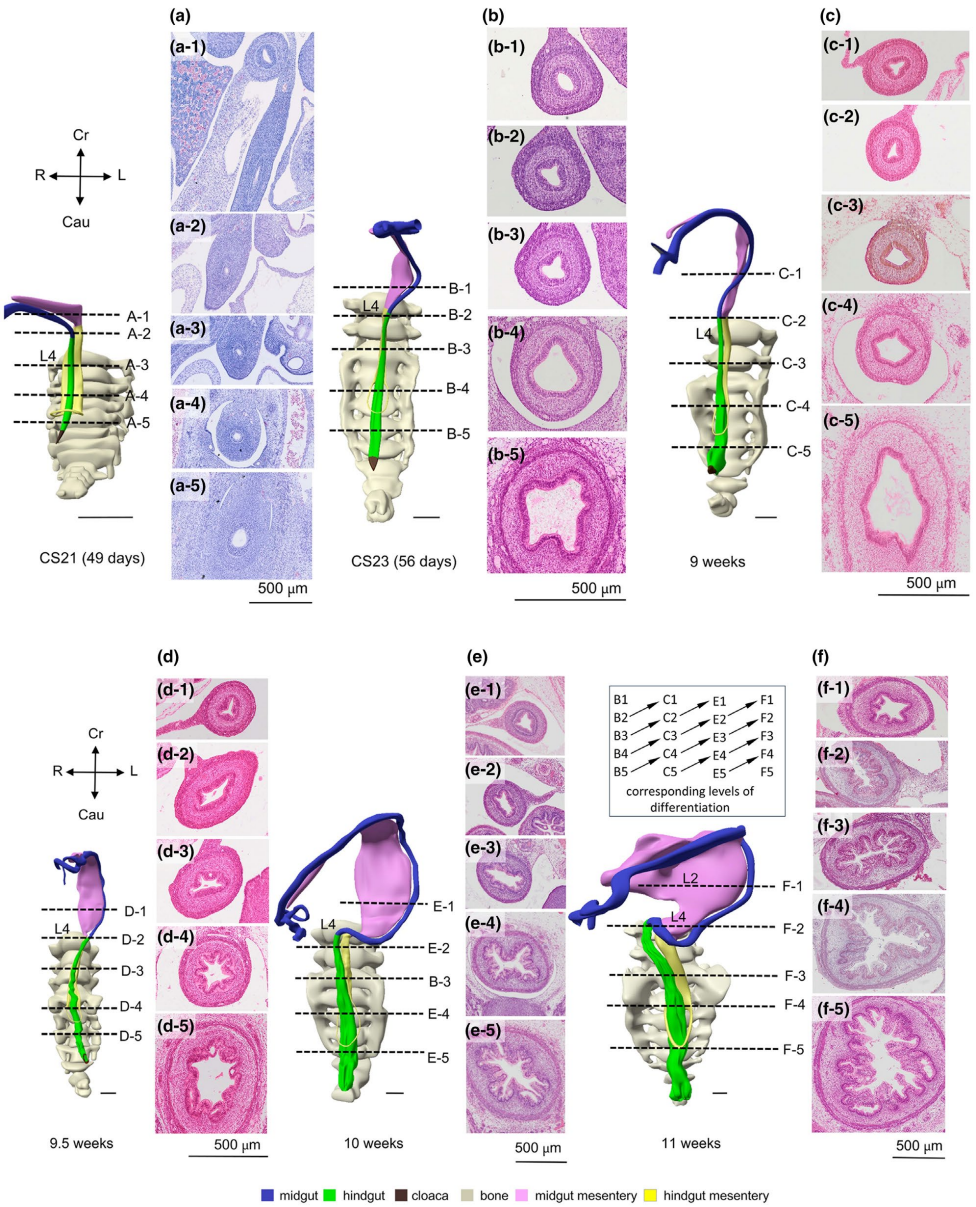
The differentiation of the colon and rectum. Ventral views at 7 (panel a), 8 (panel b), 9 (panel c), 9.5 (panel d), 10 (panel e) and 11 weeks (panel f) of development show the chronological and caudal‐to‐cranial gradients in differentiation of the wall of the colon and rectum. The transverse histological sections of the gut are localized at the levels indicated by the dashed lines (panels a1–a5 and f1–f5). The lumen of both colon and rectum is tubular until 8 weeks, and then begins to form folds. These folds develop, from caudal to cranial, a more complex folding pattern and a gradient in differentiation of its mucosal cells. Differentiation of the mucosa was assessed by the appearance and density of goblet cells. The boxed table between panels e and f provides a graphical interpretation of the decreasing degree of differentiation as a function of cranial migration and time. The shape of the epithelial surface in the top 2 rows of sections hardly changes between 9 and 10 weeks, suggesting that the caudo‐cranial differentiation gradient ends near the recto–sigmoid junction. Bars = 1 mm.

Until the end of the 7th week of development, a single layer of columnar epithelium covers the surface of the hindgut (Figure 12a). By the end of the 8th week, the lumen of the hindgut assumes a triangular shape, which is more pronounced caudally than cranially (Figure 12b). In the most caudal part, the triangle assumes a more complex, polygonal shape. This configuration moves cranially in the 9th week (Figure 12c). After the hernial return at 9.5 weeks, the mucosal wall of what we can now name the rectum starts to produce villi (Figure 12d). The degree of branching and histological maturation of these villi is best developed caudally. The complexity of the villar trees further increases during the 10th week, when secondary and tertiary villi form (Figure 12e,f). The images (Figure 12) clearly show that differentiation in the rectal mucosa progresses chronologically and from caudal to cranial. These two determinants of the mucosal differentiation process are shown schematically between columns e and f of Figure 12: each week, a certain degree of differentiation is found one step more cranially (arrows). The smooth‐muscle layers of the hindgut become identifiable at 8 weeks and also show a caudal‐to‐cranial gradient in development. At 9.5 weeks, the smooth‐muscle wall of the hindgut starts to form a circular and longitudinal layer in its most caudal part (Figure 12d). This differentiation marker has moved cranially in the 11‐week‐old foetus (Figure 12f). The typical nerve network in the muscular layer of the rectum also becomes visible at 8 weeks. It rapidly expands cranially in the 9‐week‐old foetus. These data show that all these differentiation markers move from caudal to cranial, and also that innervation precedes mucosal and smooth‐muscle layer differentiation.

## 4 DISCUSSION

### 4.1 The developmental history of the colic part of the midgut

The ‘definitive’ midgut is the part of the gut that develops a long and thin mesentery that allows its looping and transient herniation into the umbilical coelom between 5 and 9.5 weeks of development (Soffers et al., 2015). Of note, the stems of the primary loop (the jejunum and the descending limb of the colon) do not leave the abdominal cavity during the herniation. The post‐caecal gut with a thin mesentery becomes identifiable only at ~5 weeks of development and is initially still very short. At 10 weeks, the caudal transition from a thin to a thick mesentery localizes to the rectosigmoid junction. At this stage, the rectosigmoid junction is recognizable by its bend at the entrance into the pelvic peritoneal cavity, the change from a thin to a thick mesentery, and by its change in the degree of epithelial differentiation. The taeniae and haustra coli which are the histologically most reliable criteria for the colon (Massalou et al., 2018) only begin to develop at 12–13 weeks (Malas et al., 2004; Pace, 1971). The trunk of the IMA is a convenient pointing tool that reliably identifies the position of the thin‐to‐thick mesenteric junction as early as 6 weeks of development. The right‐sided, ascending limb of the colon, which herniates, becomes affixed to the duodenum, stomach and dorsal pancreas almost immediately after its return from the umbilical hernia. This fixation implies that the midgut‐to‐hindgut junction localizes further distally. While the arterial arcade that forms between the SMA and IMA at 10 weeks forms, marks the position of the midgut‐to‐hindgut junction in the vascular model (Douard et al., 2006; Frazer & Robbins, 1915; Hunter, 1928; Pernkopf, 1928), it did not leave any recognizable landmark on the colon. Finally, we observed that differentiation in the rectum, like that in the adjacent vagina (Hülsman et al., 2024b), but unlike the adjacent sigmoid colon, proceeds from caudal to cranial. The resumption of colic growth (Figure 10) and mucosal differentiation (Figure 12) at 10–11 weeks suggests that the hernial return facilitates these processes.

### 4.2 The superior and inferior mesenteric arteries define the cranial and caudal boundaries of the midgut

The midgut that we studied is located between the caudal foregut and hindgut and becomes identifiable at CS14 (~4.5 weeks). It then experiences a growth spurt that results in forming its ‘primary’ loop, but simultaneously acquires a markedly thinner mesentery than the caudal foregut and hindgut (Frazer & Robbins, 1915; Hunter, 1928; Pernkopf, 1928; Yokoh, 1970). Previously, we (Hikspoors et al., 2019; Soffers et al., 2015) and others (e.g., Douard et al., 2006; Pernkopf, 1928) described this thin mesentery to develop around the SMA, but in retrospect, we have to conclude that the root of the SMA occupies the caudal margin of the thick caudal foregut mesentery (Figure 1). Similarly, the trunk of the IMA, which becomes identifiable at CS15, is found in the cranial margin of the thick hindgut mesentery. Based on these topographical data, we conclude that the SMA and IMA do not vascularize the mid‐ and hindgut, respectively, but define the cranial and caudal boundaries of the midgut (Figures 1 and S3).

### 4.3 Intestinal growth is fastest in the midgut

A change in the definition of the boundary between the midgut and the hindgut affects their previously published growth measurements. We, therefore, reanalysed the available data of human intestinal growth. Both Mall (Mall, 1898, 1899) and Pernkopf (Pernkopf, 1925, 1928) reported exponential growth in what they defined as the small (duodenum, jejunum and ileum) and the large intestines (colon and rectum), with growth in the small intestine being >2‐fold bigger than that in the large intestine. Pernkopf's (1925) text indicates that the small intestine is the midgut (‘Mitteldarm’) in his 1928 text, suggesting they are identical. The components of his ‘Enddarm’ are the entire colon and the rectum. Harris' measurements also apply to the entire colon and rectum (Harris et al., 1976). Pernkopf's and Harris' length measurements agree (Figure S1A). Of biological interest, Pernkopf's data show a biphasic growth rate in both parts of the gut, with a major slow‐down of growth at 6–7 weeks, when herniation starts (Figure S1A). He further showed slower growth in the duodenum than the jejunum and ileum. We showed slower growth in the hindgut (rectum) than in the colon (Figure 10). Because Harris showed that the post‐splenic *plus* rectal growth exceeded that of the pre‐splenic colon by ~70% (Figure S1B; Harris et al., 1976), the growth of the post‐splenic colon *without* the rectum probably exceeds that of the pre‐splenic colon by ~2‐fold. Arsenault and Ménard described that DNA synthesis in human foetuses was lowest in the stomach and oesophagus, and ~2‐fold higher in the jejunum and colon. In all organs, DNA incorporation was highest at 8–10 weeks, the beginning of their measurements, and had declined ~2‐fold at 14–16 weeks (Figure S1; Arsenault & Menard, 1988; Arsenault & Ménard, 1987, 1989). The available data, therefore, still reflect fastest growth in the herniating portion of the gut.

### 4.4 The small‐intestinal and colic mesenteries differ

The mesenteries of the small intestine and the colon in adult humans differ in that the mesenteric root of the small intestine is short compared to that of the colon, which encircles almost the entire periphery (upper right, upper left and lower left) of the abdomen. The greater length of the colic than the small‐intestinal mesentery root is remarkable because the small intestine is ~5‐fold longer than the colon. The difference is explained by the secondary looping of the small intestine (Ishida et al., 2025; Soffers et al., 2015), while retaining its original relatively short dorsal mesentery. In contrast, the colon and its mesentery do not develop secondary loops.

### 4.5 The position of the flexures of the colon

The human foetal colon has, like most mammals, ascending or pre‐splenic and descending or post‐splenic limbs that are connected by an initially blunt ‘colic bend’ (Frazer & Robbins, 1915; Hunter, 1928; Pernkopf, 1928) or ‘colic angle’ (Frazer & Robbins, 1915; Hikspoors et al., 2019; Hunter, 1928; Pernkopf, 1928; Figures 10, S8 and S9). During the hernial period, the ascending and descending colic limbs occupy a paramedian sagittal plane on the left side of the stomach (Mall, 1898). After hernial return, the caecum occupies a fixed position on the intercristal line, which is better known as McBurney's point (Fitzgerald et al., 1971; Fröber et al., 1991; Harris et al., 1976; Hunter, 1928). As a result, the colon acquires a near‐coronal position in the abdomen. Meanwhile, the descending post‐splenic limb hardly changes in position. The ascending limb adheres to the ventral wall of the duodenum, stomach and dorsal pancreas almost immediately after hernial return (present study; Figures S8 and S9). This prompt ‘freezing’ of the topographic relations upon return rules out a pre‐splenic position of the midgut‐to‐hindgut position. In agreement, Harris demonstrated that the splenic flexure and the sigmoid bend of the colon attain their characteristic shape and position almost immediately after hernial return (Harris et al., 1976).

The splenic bend and the descending colon are fixed to the diaphragm and the left‐sided dorsal body wall between ~13 and ~17 weeks of development as the phrenicocolic ligament (Fröber et al., 1991) or as a fascia of fusion with the dorsal peritoneal wall (Congdon et al., 1942; Toldt, 1879). The ileocecal region becomes fixed at ~17 weeks only (Fröber et al., 1991). In humans, the pre‐splenic limb becomes subdivided into an ascending and a transverse colon (Pernkopf, 1928). Some investigators see a hepatic flexure develop after ~17 weeks of development (Frazer & Robbins, 1915; Hunter, 1928; Pernkopf, 1928), whereas others argue that this flexure is not unambiguously identifiable until after birth (Fitzgerald et al., 1971), subsequent to a reduction of the prenatally larger left than right liver lobe (Emery, 1963).

### 4.6 The (thick) mesorectum is a ‘real’ mesentery

In the early embryo (5–8 weeks of development), the intra‐pelvic part of the rectum is surrounded on three sides by coelom (Hikspoors et al., 2019), which qualifies the rectum as having a ‘real’ mesentery at this stage. The ‘pelvic peritoneal pouch’ reaches as far down as the muscular pelvic floor (Kruepunga et al., 2020), but it gradually disappears in the third and fourth months, along with the appearance of other secondary mesenteries in the body (such as those of the duodenum and pancreas; Figure 11; Liebermann‐Meffert, 1970; Priesching, 1957; Toldt, 1879), ascending and descending colon (Toldt, 1879) and bladder (Cunéo & Veau, 1899). These processes are reported to take place between 9–11 and 11–16 weeks, respectively (Liebermann‐Meffert, 1970; Priesching, 1957; Toldt, 1879). It has to be kept in mind that the transient ‘real’ mesentery of the rectum differs from the perirectal fat sheath that is surgically known as the ‘mesorectum’ (Heald & Moran, 1998).

### 4.7 The appearance of the first arterial arcade in the colon

Our re‐appraisal of the position of the SMA and IMA as arteries in the cranial and caudal margins of the primary loop of the midgut and its dorsal mesentery implies that these vessels will form the first arterial arcade in the dorsal mesentery. The formation of the arterial arcades is often explained by Tandler's hypothesis that they derive from a largely hypothetical ‘Langsanastomose’ (anterior or ventral longitudinal anastomosis; Tandler, 1903). It is well established that the larger ventral abdominal arteries, in particular the superior mesenteric (or ‘vitelline’) artery can initially have up to 4 segmental roots at once and can experience a ‘descent’ of its root by up to 10 of these segments (Hikspoors et al., 2015; Tandler, 1903). To account for the finding that these segmental roots will eventually merge to form a single arterial trunk, Tandler posited the ventral longitudinal anastomosis as an adaptable connection between the descending ventral arterial roots and the more peripheral parts of these vessels that have fixed positions due to their integration in their tissue environment. Our study shows, instead, that the peripheral branches of the SMA and IMA vessels begin to approach each other and form an arcade only after the return of the corresponding parts of the gut into the abdominal cavity (Figures 9, S7 and S8). In our view, there is no supporting evidence for Tandler's model (Douard et al., 2006; Geboes et al., 2001).

The vascular anatomy of the splenic flexure varies among individuals, with a third of the population having a connection between the SMA and IMA at the level of the trunks of both vessels (the ‘intermesenteric trunk’) and the remaining two‐thirds having a more peripheral connection between the middle and left colic arteries. In our 10‐week specimen, both vessels had formed (Figures 9 and S8). However, the most peripheral arterial arch, known as the ‘marginal artery’ of Drummond (Bruzzi et al., 2019; Murono et al., 2022), is not yet present in the splenic bend at 10 weeks (Figure 9).

### 4.8 The caudal‐to‐cranial progress in differentiation is typical for the pelvic organs

Between the 8th and 10th week, the hindgut undergoes significant changes due to the remodelling of the dorsal cloaca (Kruepunga et al., 2018), with ridges, folds, villi and glands appearing sequentially in this region (Figure 12). As the glands develop further, the villi follow (Bell & Williams, 1982; Colony et al., 1989; Helander, 1973; Lev & Orlic, 1974), as does the differentiation of the circular and longitudinal smooth muscles. The rectal villi remain present for a considerable period of embryonic development, but disappear before birth (Johnson, 1914). The extrinsic autonomic innervation also appears later in the colon than in the rectum (Kruepunga et al., 2020). The cranio‐caudal gradient in differentiation of the (derivatives of the) midgut and the caudo‐cranial gradient in the rectum (Figure 12) meet somewhere near the rectosigmoid junction. The mucosa of the vagina also differentiates in a caudal‐to‐cranial direction: the original epithelial lining of the uterus and vagina derives from the paramesonephric (Müllerian) ducts, whereas the definitive vaginal epithelium is now generally believed to have an origin in the epithelium of the urogenital sinus that migrates cranially from its origin in the Müllerian tubercle (Davis & Pearl, 1938; Hülsman et al., 2024b; Koff, 1933; Robboy et al., 2017). Much less is known about a gradient in the differentiation of the urinary system, but the differentiation of the epithelial (Saizonou et al., 2024) and smooth‐muscle (Baker & Gomez, 1998) layers of the bladder and ureters also proceeds from caudal to cranial. The differentiation of the epithelium of the urinary collecting system differentiates from the renal pelvis to the renal periphery (Saizonou et al., 2024). The central‐to‐peripheral direction of the epithelial differentiation concurs with that seen in other branching epithelial organs.

It is not known what signal induces the caudo‐cranial gradient in differentiation. The timeline of the arrival of the respective extrinsic and enteric autonomic neurons in the inferior pelvic plexus and hindgut is similar (Fu et al., 2004; Kruepunga et al., 2020; Okamoto & Ueda, 1967). The intrinsic enteric neurons of the hindgut were long suspected (Young & Ciampoli, 1998) and recently shown to be of vagal neural‐crest origin in mice (Yu et al., 2024). Of interest, the nitrergic neurons among these cells are far more numerous caudally than cranially in the rectum and, although their number declines with age, retain this caudocranial gradient in density between the second trimester of pregnancy (Román et al., 2004) and several years after birth (Wester et al., 1999).

## 5 CONCLUSION

The used landmarks allowed us to identify the rectosigmoid junction as the junction between the midgut and hindgut in the human embryo. The rectum is, therefore, the sole descendant of the hindgut. In contrast to a junction of the midgut–hindgut that is based on the position of the first arterial arcade, the junction based on the thin–thick mesenterial criterion also corresponds with the structural differences between the sigmoid colon and rectum.

## AUTHOR CONTRIBUTIONS

H.G. participated in data collection, analysis and visualization, wrote the manuscript and was responsible for formatting the figures. N.K., G.M., S.E.K and J.H. participated in data analysis and interpretation and edited the manuscript. W.H.L. conceived the study and supervised the interpretation of the data and the preparation of the manuscript.

## Supporting information


Data S1.

Figure S1.

Figure S2.

Figure S3.

Figure S4.

Figure S5.

Figure S6.

Figure S7.

Figure S8.

Figure S9.

## Data Availability

All data is original data and can be shared.
