# Supplementary material for: The junction between the midgut and hindgut co‐localizes with the rectosigmoid junction
Source: J Anat. 2025 Nov 14;249(1):33–53. doi: 10.1111/joa.70070 (PMC13238873; doi:10.1111/joa.70070)
Supplement: Supplementary file 1 — Data S1. [file JOA-249-33-s008.docx]

**Supplementary Figures**

**Figure S1. Growth of the foetal gut.**

Pernkopf’s and Harris’ measurements of the small and the large intestine *plus* rectum (Pernkopf, 1928) (Harris et al., 1976). Pernkopf’s data of the colon *plus* rectum (green symbols) are similar to those of Harris (yellow symbols; panel A)). All intestines grow exponentially (note semilogarithmic Y-axis). The growth of the small and the large intestines *plus* rectum can be divided into two phases: rapid growth up to ~50 mm CRL (~9 weeks), followed by slower growth. The small intestine grows almost 2-fold faster than the large intestine *plus* rectum during the 7^th^ and 8^th^ weeks, but growth rates become similar thereafter. In panel B, separation of Harris’ data shows that the ascending limb of the foetal colon (caecum to splenic flexure; dashed purple line) grows slightly slower than the descending limb *plus* rectum (from the splenic flexure to the anal sphincter; dashed orange line). Panel C shows thymidine incorporation into the epithelium of the oesophagus (Arsenault and Menard, 1988), stomach (Ménard and Arsenault, 1990), jejunum (Arsenault and Ménard, 1987) and colon (Arsenault and Ménard, 1989). Thymidine incorporation declined with age in all 4 organs but was higher in the jejunum and colon (organs with a thin mesentery) than in the stomach or oesophagus (organs with an initially thick mesentery).

**3D-PDFs**

**3D-PDF user instructions**

To view the interactive 3D-PDF to its full potential you need to download the 3D-PDF to your computer (a 3D-PDF file can be opened on any computer as long as it contains the Adobe PDF reader). To activate the 3D-PDF you need to click on the model. A toolbar appears at the top of the screen. Under options, you must state that you trust this document. If you then click on the model, a toolbar appears on the left side of your screen that includes the option ‘model tree’. The model tree displays a material list of structures in the upper box and preset viewing options in the lower box. The list of visible structures can be modified by marking or unmarking a structure. We advise adding structures to an initially basic configuration rather than the other way around: “dress, do not undress”. To manipulate the reconstruction, press the left mouse button to rotate it, the scroll button to zoom in or out, and the left and right mouse buttons simultaneously to move the embryo across the screen. The colour code in all Figures is identical (shown in Table 2), and all structures are listed by the same name in the respective ‘model trees’. To best see the position of the arterial vessels inside the dorsal mesentery, the reader is advised to use the 3D-PDFs, select the thin dorsal mesentery, the arterial vessels and, if so wished, another structure. Then right-click the “mesentery” label in the model tree and click on “transparent”. By moving the model, the topographic relation between vessels and mesentery can be observed from all sides.

**Figure S2: Interactive 3D-PDF of the abdomino-pelvic region of a 4.5-weeks** **human embryo (CS14; S6502).**

At CS14, the ventral mesentery of the foregut serves as a key anatomical landmark for distinguishing the boundary between the caudal foregut (grey) and midgut (blue). The junction of the midgut and hindgut (green) corresponds with the transition of a thin (pink) and a thick (yellow) dorsal mesentery. The yolk stalk is positioned at the ventral apex of the midgut. Rib of cube: 1 mm.

**Figure S3: Interactive 3D-PDF of the abdomino-pelvic pelvic region of a ~5-weeks human embryo (CS15; S2213).**

At CS15, the trunk of the inferior mesenteric artery (IMA) is found at the junction of the thin mesentery of the midgut and the much thicker mesentery of the hindgut. The branching sites of the left colic artery (LCA) and the superior rectal artery (SRA) correspond precisely with the midgut-hindgut boundary. The yolk stalk at the ventral apex of the midgut is withering. Rib of cube: 1 mm.

**Figure S4: Interactive 3D-PDF of the abdomino-pelvic region of a ~6.5-weeks human embryo (CS18-late; S4430).**

At CS18, the primary midgut loop has fully formed. The caecum is located in the ascending (returning) limb of the primary loop. The bifurcation of the IMA corresponds with the midgut-hindgut junction. Rib of cube: 1 mm.

**Figure S5: Interactive 3D-PDF of the abdomino-pelvic region of an ~8-weeks human embryo (CS23; S48).**

With the exception of the developing jejunum and ascending colon, the intestinal loops are found in the umbilical hernia. The trunk of the IMA trunk has adopted a craniocaudal orientation, but its first branching point still identifies the midgut-hindgut junction. Rib of cube: 1 mm.

**Figure S6: Interactive 3D-PDF of the abdomino-pelvic region of a ~9.0-weeks human foetus (S89).**

The midgut returns into the abdominal cavity. The mesentery of the colon partially attaches to the mesenteric block (light grey) of the cranial midgut, while the distal mesentery of the colon connects directly to the dorsal abdominal wall via the thin mesentery and continues into the thick mesenteric root of the hindgut. Rib of cube: 100 mm.

**Figure S7: Interactive 3D-PDF of the abdomino-pelvic region of a ~9.5-weeks human foetus (S57).**

The caecum has re-entered the abdominal cavity but still occupies a ventral midline position. The IMA has branched into the left colic artery, superior rectal artery, and a sigmoid artery. Rib of cube: 1 mm.

**Figure S8: Interactive 3D-PDF of the abdomino-pelvic region of a ~10-weeks human foetus (S4908).**

The caecum has shifted from the ventral midline to the right abdomen. The splenic bend has formed in the upper left abdomen, and the descending colon has extended sinistro-caudally. At the midgut-hindgut junction, the sigmoid loop has started to form. The branches of the superior and inferior mesenteric arteries begin to anastomose, forming the first arterial arcade of the colon. The midgut-hindgut connection (rectosigmoid junction) begins to descend from the L4 vertebral level. Rib of cube: 1 mm.

**Figure S9: Interactive 3D-PDF of the abdomino-pelvic region of an ~11-weeks human foetus (S1744).**

The colic mesentery attaches to the proximal duodenum, the ventral stomach, and the pancreatic tail. Rib of cube: 1 mm.

Arsenault P, Menard D. 1988. Autoradiographic localization of [3H]‐thymidine incorporation in developing human esophagus. The Anatomical Record 220:313-317.

Arsenault P, Ménard D. 1987. Cell proliferation in developing human jejunum. Neonatology 51:297-304.

Arsenault P, Ménard D. 1989. Cell proliferation during morphogenesis of the human colon. Neonatology 55:137-142.

Harris P, Jones P, Robertson C. 1976. A radiological study of morphology and growth in the human fetal colon. The British Journal of Radiology 49:316-320.

Ménard D, Arsenault P. 1990. Cell proliferation in developing human stomach. Anatomy and embryology 182:509-516.

Pernkopf E. 1928. Die Entwicklung der Form des Magen-Darm-Kanales beim Menschen: II. Teil, 3. Abschnitt Die weitere Ausbildung des Enddarmes, insbesondere des Blinddarmes und der Kolonteile. Zeitschrift für Anatomie und Entwicklungsgeschichte 85:1-130.
