# Supplementary figures and images for: The junction between the midgut and hindgut co‐localizes with the rectosigmoid junction
Source: J Anat. 2025 Nov 14;249(1):33–53. doi: 10.1111/joa.70070 (PMC13238873; doi:10.1111/joa.70070)

Carnegie Stage 14

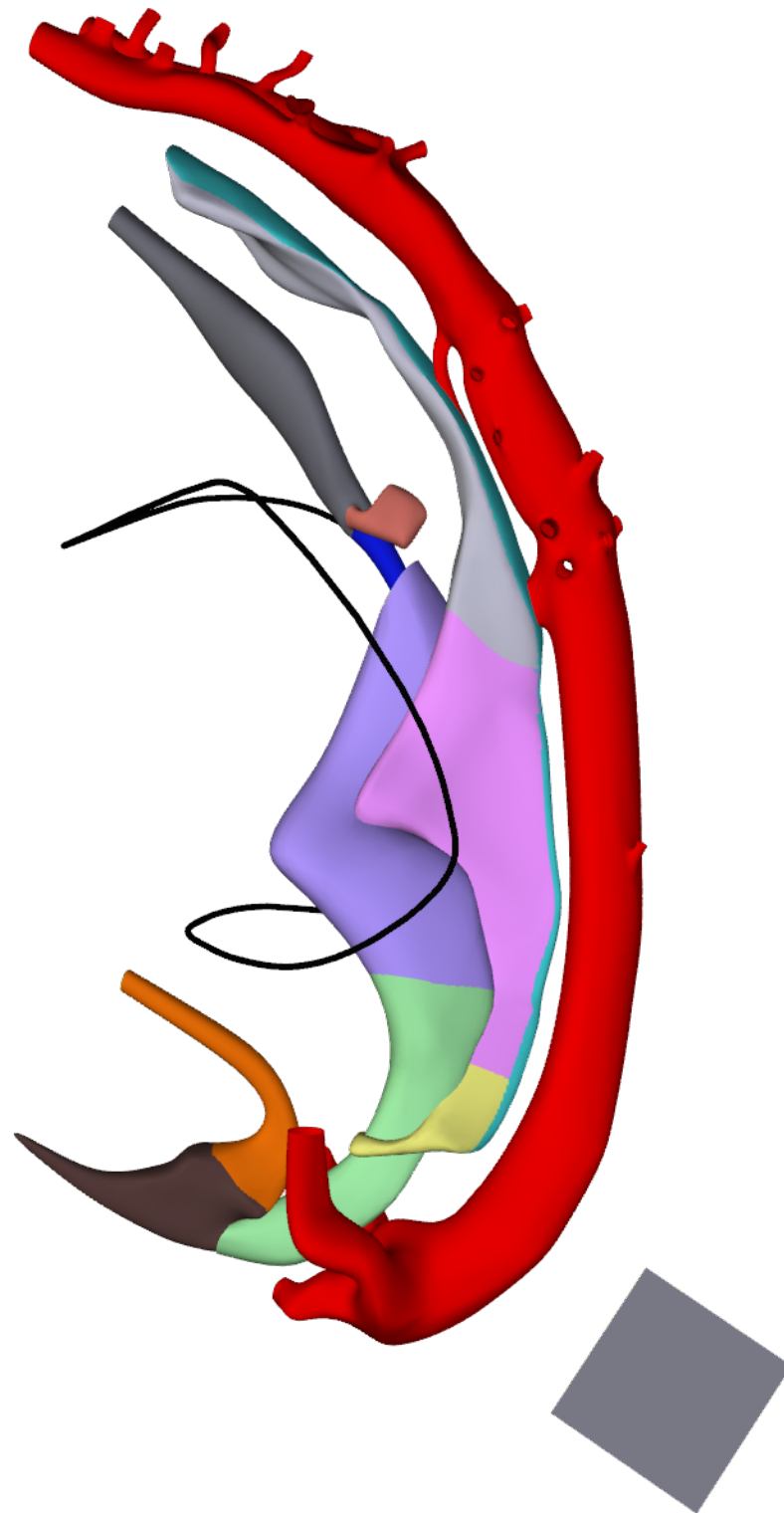

H. Gao, et al. 2025

Supplement: Supplementary file 3 — Figure S2. [file JOA-249-33-s009.pdf]

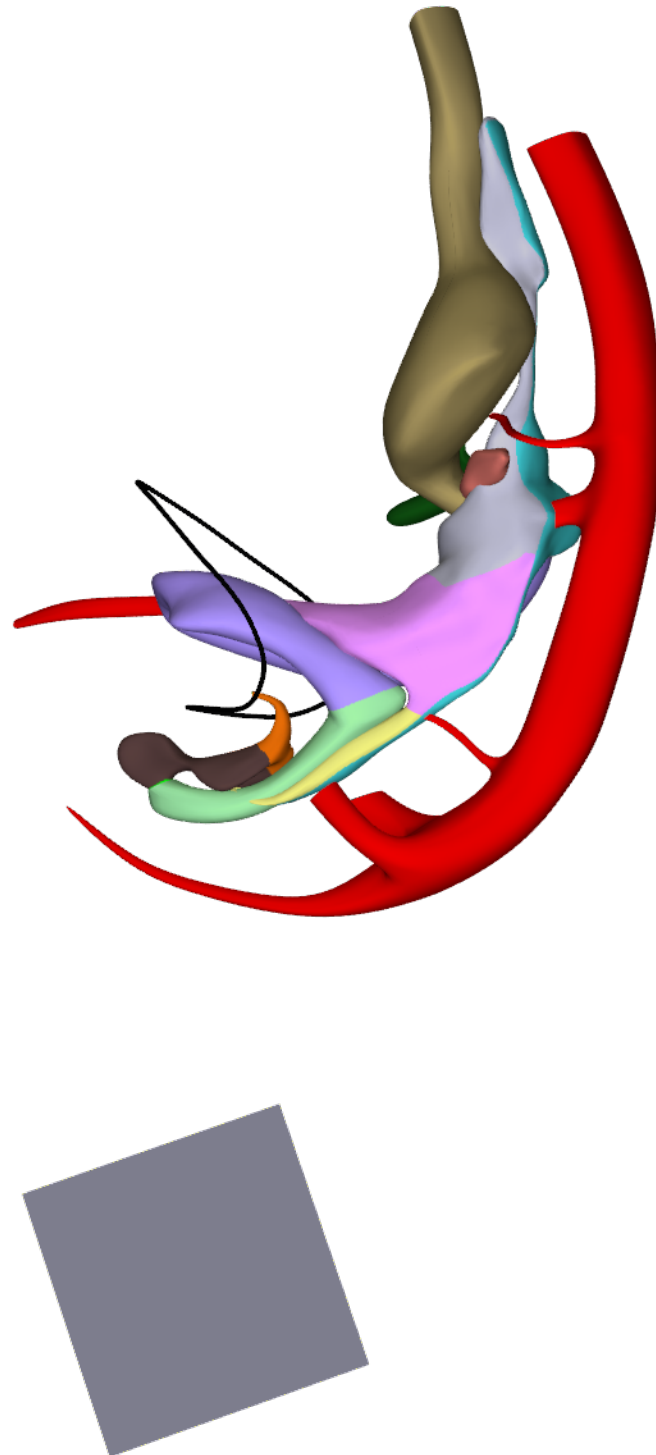

Supplement: Supplementary file 4 — Figure S3. [file JOA-249-33-s010.pdf]

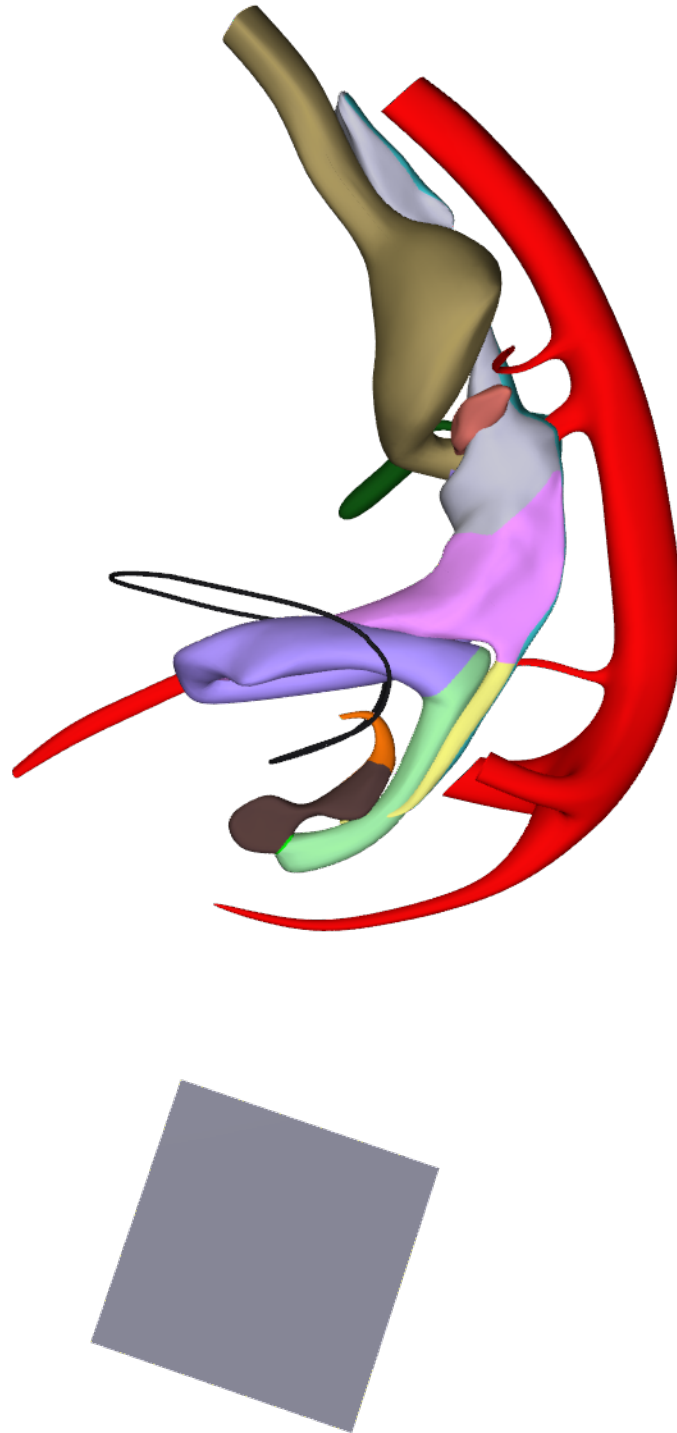

Supplement: Supplementary file 5 — Figure S4. [file JOA-249-33-s007.pdf]

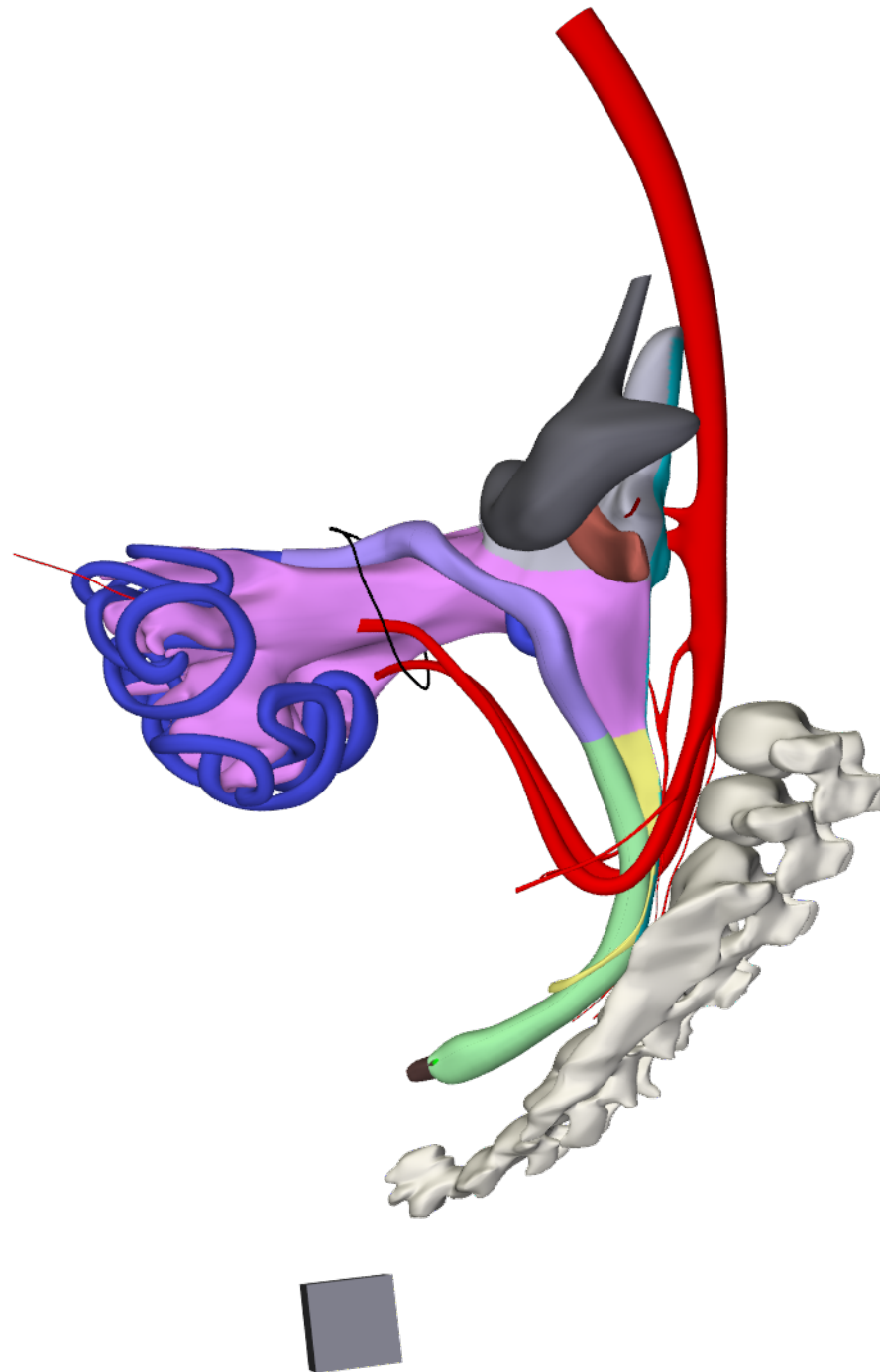

Supplement: Supplementary file 6 — Figure S5. [file JOA-249-33-s005.pdf]

9.0 weeks

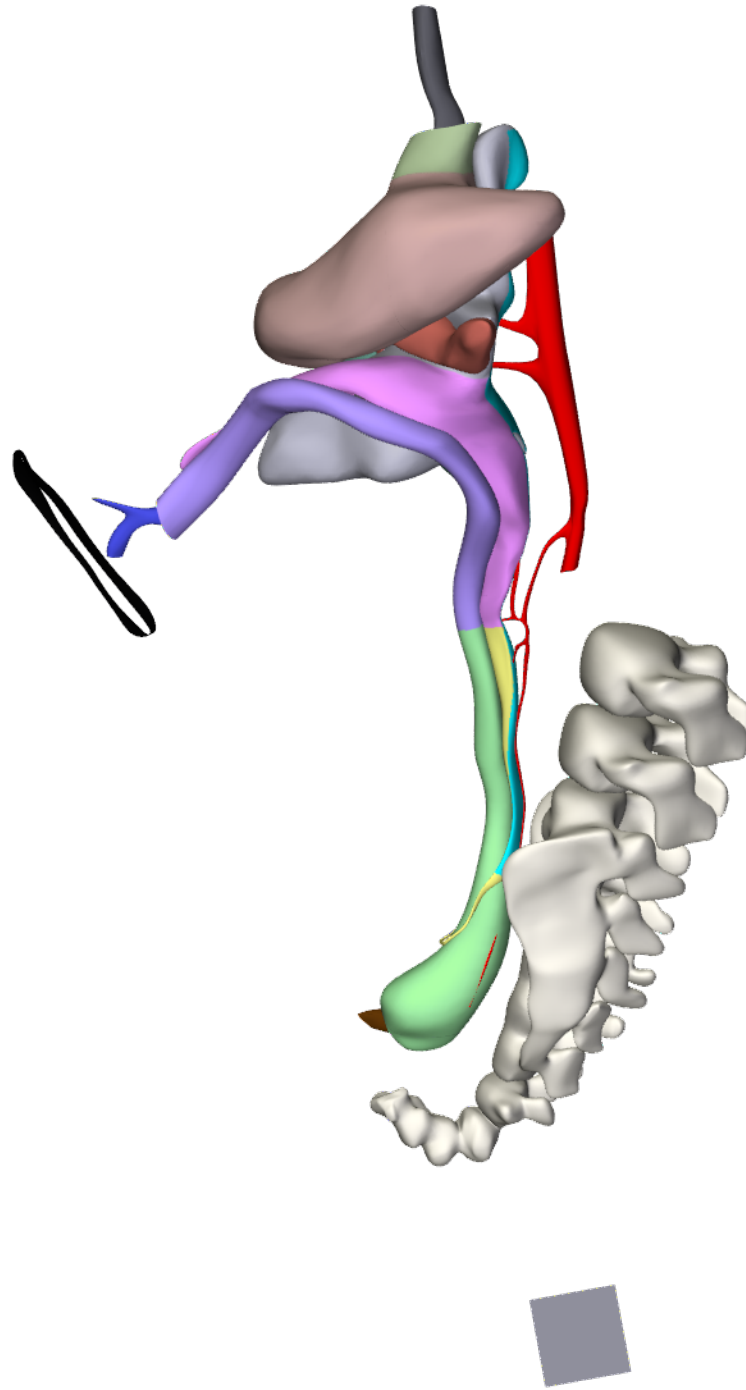

Supplement: Supplementary file 7 — Figure S6. [file JOA-249-33-s001.pdf]

9.5 weeks late

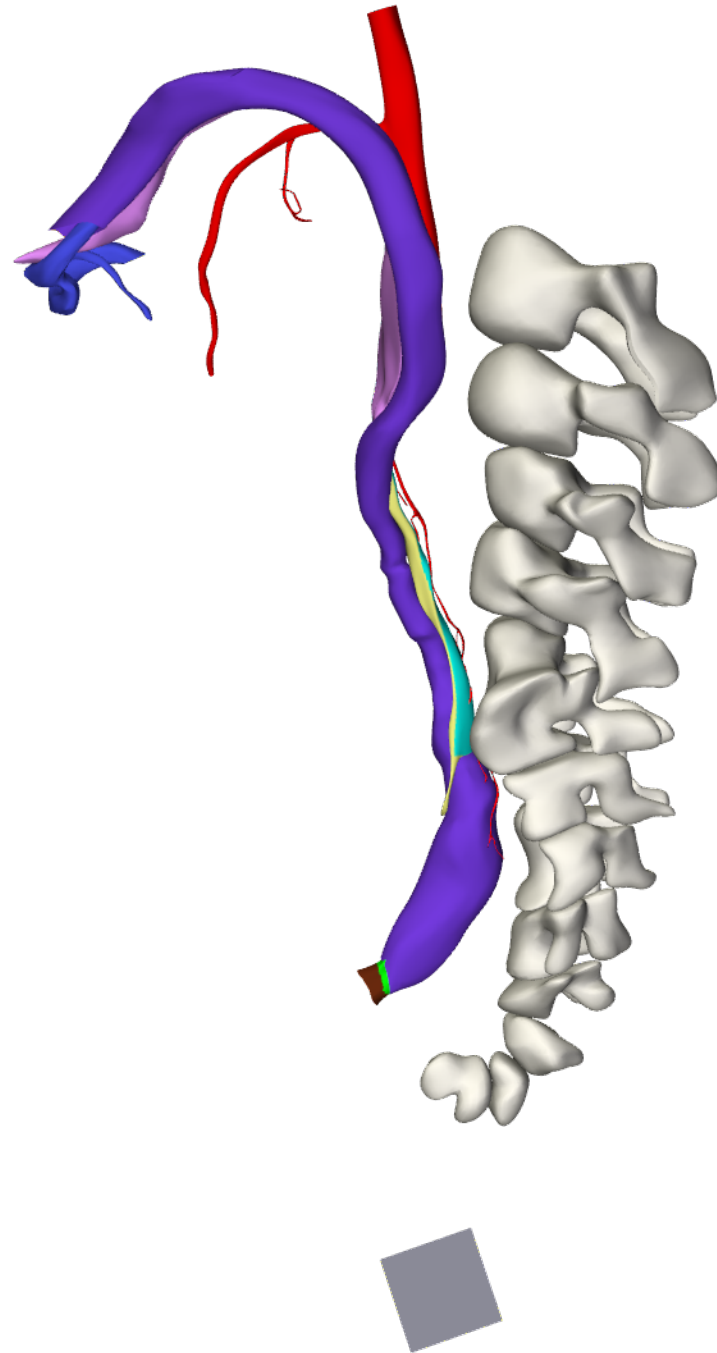

Supplement: Supplementary file 8 — Figure S7. [file JOA-249-33-s002.pdf]

10 weeks

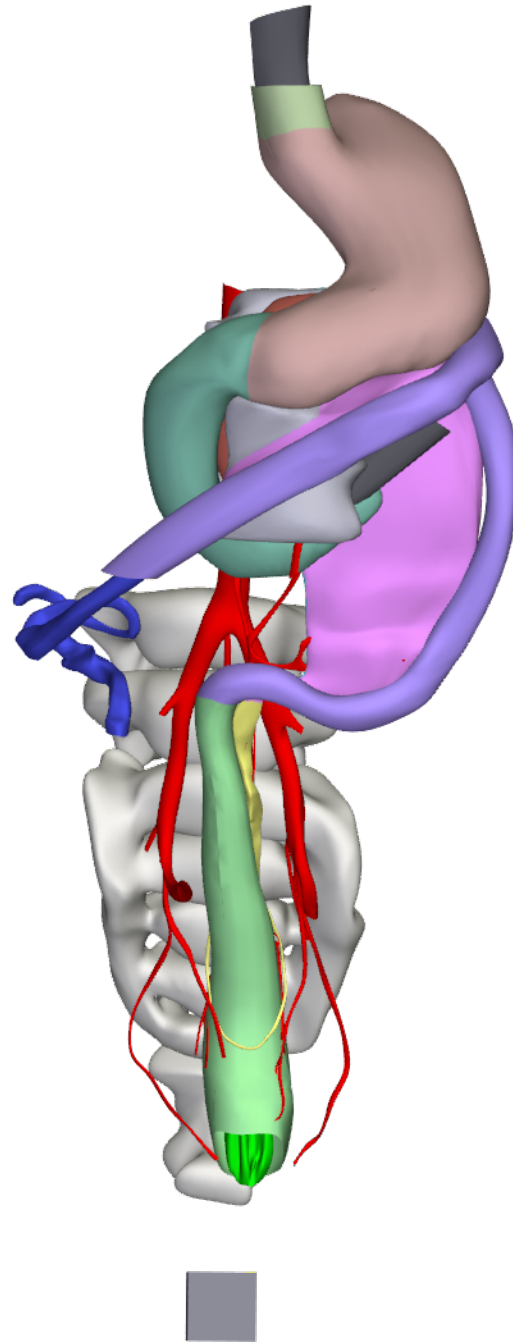

Supplement: Supplementary file 9 — Figure S8. [file JOA-249-33-s003.pdf]

11 weeks

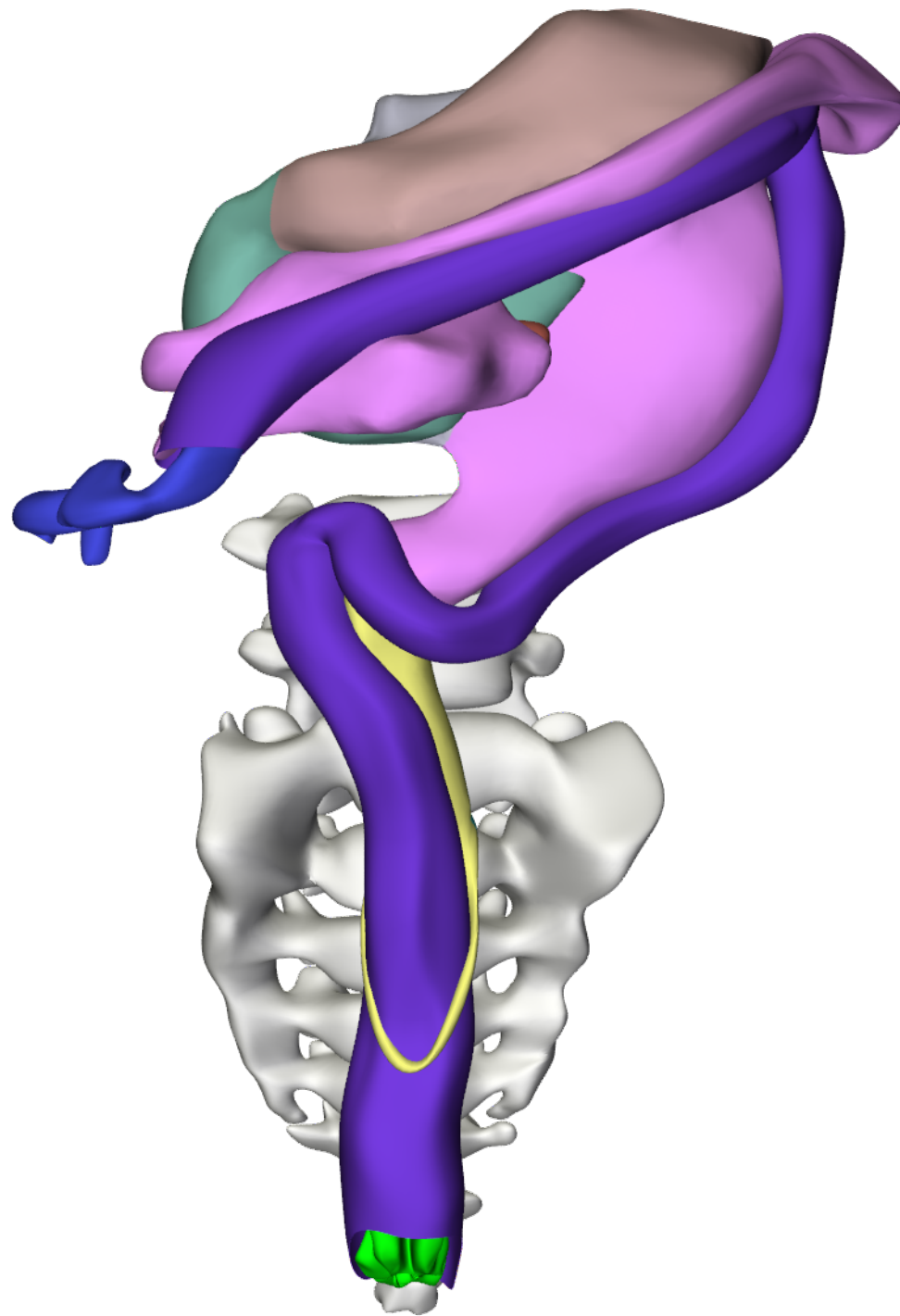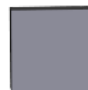

Supplement: Supplementary file 10 — Figure S9. [file JOA-249-33-s004.pdf]
